# Plant Size as Determinant of Species Richness of Herbivores, Natural Enemies and Pollinators across 21 Brassicaceae Species

**DOI:** 10.1371/journal.pone.0135928

**Published:** 2015-08-20

**Authors:** Hella Schlinkert, Catrin Westphal, Yann Clough, Zoltán László, Martin Ludwig, Teja Tscharntke

**Affiliations:** 1 Agroecology, Georg-August-University Göttingen, Göttingen, Germany; 2 Centre for Environmental and Climate Research, Lund, Sweden; 3 Hungarian Department of Biology and Ecology, Babes-Bolyai University, Cluj-Napoca, Romania; 4 Institute of Horticultural Production Systems, Department Phytomedicine, Leibniz University Hannover, Hannover, Germany; UMR INRA/INSA, BF2I, FRANCE

## Abstract

Large plants are often more conspicuous and more attractive for associated animals than small plants, e.g. due to their wider range of resources. Therefore, plant size can positively affect species richness of associated animals, as shown for single groups of herbivores, but studies usually consider intraspecific size differences of plants in unstandardised environments. As comprehensive tests of interspecific plant size differences under standardised conditions are missing so far, we investigated effects of plant size on species richness of all associated arthropods using a common garden experiment with 21 Brassicaceae species covering a broad interspecific plant size gradient from 10 to 130 cm height. We recorded plant associated ecto- and endophagous herbivores, their natural enemies and pollinators on and in each aboveground plant organ, i.e. flowers, fruits, leaves and stems. Plant size (measured as height from the ground), the number of different plant organ entities and their biomass were assessed. Increasing plant size led to increased species richness of associated herbivores, natural enemies and pollinating insects. This pattern was found for ectophagous and endophagous herbivores, their natural enemies, as well as for herbivores associated with leaves and fruits and their natural enemies, independently of the additional positive effects of resource availability (i.e. organ biomass or number of entities and, regarding natural enemies, herbivore species richness). We found a lower R^2^ for pollinators compared to herbivores and natural enemies, probably caused by the high importance of flower characteristics for pollinator species richness besides plant size. Overall, the increase in plant height from 10 to 130 cm led to a 2.7-fold increase in predicted total arthropod species richness. In conclusion, plant size is a comprehensive driver of species richness of the plant associated arthropods, including pollinators, herbivores and their natural enemies, whether they are endophagous or ectophagous or associated with leaves or fruits.

## Introduction

The body size of organisms has a large impact in physiological, genetic and ecological contexts [1]. Large body size is often related to greater dispersal ability, enhanced competitiveness for resources [2,3] and can affect communities of associated organisms [4,5]. Differences in plant size can lead to differences in conspicuousness (being apparent) and in attractiveness (arousing interest) of plants for associated organisms. Not only visual but also chemical conspicuousness of large plants can be high, as they provide large surface area for emission of volatile organic compounds, which are often perceived by plant associated organisms (apparency hypothesis [6–8]). In addition, attractiveness of large plants to associated organisms may be high as they can offer large microhabitat area, a high quantity and variety of resources and niches, high plant vigour and low levels of defensive compounds (species area relationship, resource diversity, plant vigour and growth-defense trade-off hypotheses [9–11]).

A positive correlation between plant size and species richness of associated animals has been shown in several studies [12–14], particularly with focus on single ectophagous herbivore groups. The effect of plant size on species richness of associated organisms may further depend on the modality of their association with the plant. Espírito-Santo et al. [15] showed a positive effect of plant size on species richness of ectophagous herbivores but not on species richness of endophagous herbivores, which was driven by resource availability instead but see [13,16]. Most herbivore species are specialised on certain plant organs [17] and are known to be affected by characteristics of their resource organs, such as biomass and number [18,19]. High quantity or biomass of the relevant organs may increase their attractiveness and conspicuousness to associated organisms. Additionally, the conspicuousness of the plant organs may be positively related to plant size because organs of large plants are often less hidden in the surrounding vegetation than those of small plants. Accordingly, densities of caterpillars with specialisation on leaves of *Calluna vulgaris* (L.) Hull were reported to increase with increasing intraspecific plant height [12].

Species richness of predators and parasitoids is positively affected by resource availability, namely by species richness of their prey and hosts (herbivores) [20,21]. Additionally, different plant characteristics can affect prey and host location of herbivores’ natural enemies [22,23], whereby large plants may be highly apparent prey and host habitats due to their visual and chemical appearance. This effect of plant size on natural enemy species richness may depend on whether their prey or host species are endophagous or ectophagous herbivores, since different degrees of concealment can lead to differences in prey and host location strategy [24].

Pollinators, another important group of plant associated arthropods, are influenced by flower characteristics such as number, size, colour and scent [25–28]. Additionally, they can be affected by plant size when inflorescence height increases with plant size and flowers of large plants thereby gain in conspicuousness. A positive effect of plant size has been shown for pollinator abundance or visitation rates along intraspecific plant size gradients [29–31], but not for pollinator species richness.

Most studies on the effect of plant size on species richness of associated organisms focus on single insect groups only, on intraspecific plant size gradients and often lack standardisation as they sample plants in fields with different local conditions such as soil fertility or interspecific competition, species pools and surrounding landscapes. Data collected in the field (in contrast to those resulting from standardised common garden experiments) may suffer from a bias as mean plant size increases with successional stage of the vegetation and thereby with overall biodiversity, so that larger plant species typically grow in more diverse environments [32]. One advantage of interspecific over intraspecific plant size gradients is that they can be broader without strong bias by factors such as age, nutrient availability, competitive pressure or influence of interaction partners resulting in smaller or larger individuals [e.g. 33]. Furthermore, conclusions drawn on patterns across species are of greater generality than those of intraspecific case studies.

In this study we comprehensively analyse the effect of plant size along a broad interspecific gradient on herbivores, their natural enemies and pollinators under standardised conditions. We thereby accounted, when appropriate, for organ characteristics and resource availability to disentangle their effects from those of plant size *per se*. The following hypotheses are tested: Species richness of herbivores (overall, ectophagous, endophagous, leaf and fruit associated herbivores), their respective natural enemies and pollinators increase with plant size (1). Additionally, resource quantity has a positive effect on species richness (2): species richness of natural enemies increases with prey/host availability, species richness of leaf and fruit associated herbivores and of their respective natural enemies is positively affected by biomass and number of leaves and fruits, while flower characteristics, namely number, biomass and colour, additionally contribute to the explanation of differences in pollinator species richness. The overall effects of plant size on species richness of herbivores, their natural enemies and pollinators are all positive, while these effects regarding herbivores and pollinators are more pronounced in comparison to natural enemies of herbivores, as they directly depend on the plant as resource (3).

## Materials and Methods

### Study site and sampling design

The study site was located in Göttingen (Lower Saxony, Germany; 51.5° N, 9.9° E) in a grassland with a variety of brassicaceous herbs (the land accessed is owned by the university). We chose 25 Brassicaceae species covering a plant size gradient and established a common garden experiment in summer 2010. Plant species which could not be brought to full flowering between mid-June and mid-July 2010 were excluded from the data set to avoid phenological differences in the local insect community of the study area. The remaining 21 plant species covered a plant size gradient from 12.65 cm ± 1.05 cm height (*Diplotaxis muralis* (L.) DC.) to 120.50 cm ± 2.95 cm height (*Raphanus sativus* L. *oleiformis*) (arithmetic mean ± SE). Chosen species are widespread annuals in Germany and have many features in common, such as a similar evolutionary background (as they are from the same family), the family typical flower shape, the presence of glucosinolates as secondary plant substances and the pollination ecology, since insect pollination increases their seed set [34]). The common garden experiment consisted of 100 plots with a size of 1 m^2^ and a distance of 30 cm to each other (for a photo of the experimental site see S1 Fig). Four plots per plant species were established in monoculture in a completely randomised design. All plots were fertilised once (NPK fertiliser with the ratio of 15:6:12) and regularly irrigated and weeded. Density of plants was regulated to not exceed the plot boundaries and to reach about 100% plant cover by the time of flowering. The plot based approach allowed us to focus on effects of plant size (measured as height) while controlling for effects of plant area (by the standardisation of ground surface area covered by a certain plant species). This approach further implied an inverse correlation of plant density per plot and plant size, representing a common effect under natural conditions (“self-thinning rule”, [35]).

### Arthropod surveys

Free-living arthropods were assessed on the different plant organs (flowers, fruits, stems and leaves) of five randomly chosen and individually marked plant individuals per plot once at its time of full blossom (between mid-June and mid-July 2010). Flower visiting Hymenoptera, Diptera and Lepidoptera were thereby omitted and separately sampled (see below). Parasitised animals, such as mummified aphids or cabbage moth pupae, were collected alive and parasitoids were reared.

To assess endophagous arthropods we harvested all leaves of plant individuals from one quarter of every plot, counting the respective plant individuals, and also harvested the stems and fruits of five randomly selected plant individuals per plot. The harvest of leaves and stems took place at the time of early ripening for each plot. Thereby the five individually marked plant individuals were excluded from this sampling so that they could develop pods, which we harvested at the time of full ripening of each plot in order to sample endophagous arthropods. To properly assess endophagous arthropods only, all free-living arthropods were removed from collected fruits, stems and leaves, with the exception of larvae and eggs of *Aleyrodes proletella* L., which can be easily overlooked in the field and are ecologically close to endophagous arthropods with regard to their host plant choice (their egg-laying mother chooses their host plant which they generally are not able to leave [36]). Animals from collected leaves and fruits were reared, while stems of first and second order were dissected and animals collected.

All animals (ectophagous and endophagous) were identified to species level and classified into herbivores, natural enemies and “others” based on the developmental stage (egg, larva, pupa or imago) at which animals were observed in the field. Whenever the host of a parasitoid could not be found in the sample, we used parasitoid-host relationships from literature to infer the identity of the parasitised herbivore and added it to the dataset. Species richness of herbivores and natural enemies was calculated for five plant individuals per plot, either by pooling animals of the five plant individuals or, in the case of leaf associated endophagous arthropods, by rarefying species richness to five plant individuals using the vegan-package in R [37]. Subsets of the dataset were then created for ectophagous and endophagous herbivores and their natural enemies separately, and for herbivores associated with the single plant organs and their respective natural enemies.

Flower visiting Hymenoptera, Diptera and Lepidoptera were sampled three times on every plot at its time of full blossom during a 5 min. observation period and a net 5 min. catching period (handling time not included). During the observation period, pollinators were identified as accurately as possible without disturbance, while during the catching period we caught every pollinator that could not immediately be identified to species level for later identification. Pollinators from the three runs were pooled for each plot. Pollinator abundance per plot was calculated from observation data, pollinator species richness per plot was calculated from observation and catching period data.

### Ethics Statement

Assessment of animals was carried out in accordance with the Federal Nature Conservation Act (§ 43 Abs. 8 Nr. 3), and with approval from the local nature conservation authorities (Untere Naturschutzbehörde Stadt Göttingen, Fachdienst Umwelt: Approval No. 67.2.5 Wei).

### Plant trait surveys

During the specific period of full blossom of each plot we recorded plant size (height from the ground to the top of the plant), number and size of flowers and leaves (petal length in mm, area of the lowest living leaf per plant in cm^2^) for five randomly selected plant individuals for each plot. Number and size of fruits (length times width in mm^2^) was recorded for five randomly selected plant individuals of each plot at the time of full ripening. To assess the biomass of the different organs we collected all flowers, leaves and fruits of all plant individuals in one quarter of every plot (in the case of flowers of two randomly selected plots per plant species) and counted the number of harvested plant individuals. We harvested flowers at the time of full blossom of each plot, leaves at the time of early ripening and fruits at the time of full ripening, after completing the arthropod sampling for the respective organs. Plant individuals with harvested organs were excluded from further observations, e.g. harvest of fruits was not performed on individuals whose flowers had already been collected. Collected organs were oven-dried for 48 h at 60°C before dry biomass was assessed.

Averages of plant size and size of flowers, leaves and fruits were calculated for each plot. Because herbivores and natural enemies were surveyed on five plant individuals per plot, we consequently assessed number of leaves and fruits on these individuals. Likewise, biomass of leaves and fruits per five plant individuals was calculated for every plot by multiplying by five the mean dry organ biomass per plant of the relevant plot. To combine flower data with pollinator data sampled at plot scale, we extrapolated number and biomass of flowers to plot level by extrapolating the mean value per plant individual of the relevant plot to the number of plant individuals. Flower colour was categorised as yellow or white, dependent on the plant species.

### Statistics

Linear mixed effects models implemented in the nlme R package [38] of R version 3.2.0 [39] were used to test the effects of plant size on species richness of plant associated arthropods. Plant species was included as a random effect in each model to account for the non-independence of the four plots per plant species. As response variables we used total species richness of herbivores, species richness of endophagous and ectophagous herbivores and species richness of herbivores associated with fruits and leaves of five plant individuals, species richness of their respective natural enemies and species richness of pollinators per plot. Datasets of arthropod species associated with stems and flowers consisted of many zeros and were too limited for separate analyses (for mean values and SE see results). For each analysis we used plant size as the main explanatory variable and added covariables and all two-way interactions depending on the response variable. For analyses of species richness of fruit and leaf herbivores and their natural enemies, we added the number and biomass of leaves or fruits per five plant individuals of every plot. For all analyses of natural enemy species richness, we added the relevant species richness of herbivores of every plot. For analysis of pollinator species richness, we added flower number per plot, flower biomass per plot and flower colour as covariables, involving only those plots on which flowers were harvested (2 * 21 = 42 plots instead of 4 * 21 = 84 plots for other analyses). All covariables regarding organ characteristics and resource availability were added to the models to disentangle their effects from effects of plant size and to account for potential species-specific differences in these characteristics.

As some explanatory variables of different models were not independent of each other (Table 1), we tested the variance inflation factor for every model of this study using the HH-package of R [40]. Since the variance inflation factor only slightly exceeded the value of 3 in one case (the model with natural enemy species richness of leaves as a response variable exceeding the value of 3 by 0.5), the parallel use of the explanatory variables in the models was statistically sound [41]. Organ size was correlated to several other plant characteristics and its incorportation would have raised the variance inflation factor significantly (S1 Table). We used log- or square-root-transformations of variables or standard classes of variance function structures implemented in the nlme-package of R whenever necessary to avoid heteroscedasticity and non-normal error distribution. AICc values of simplified models were compared with all possible combinations of the full model variables using the dredge function incorporated in the MuMIn package of R [42]. The models with the lowest AICc in a delta 2 range, in which model fit is assumed to be similarly good, were averaged for the sole purpose of obtaining parameter weights (also called “relative variable importance”) for every explanatory variable [43,44]. Given the covariance of explanatory variables we used parameter weights rather than p-values for the detection of variables which explain a significant part of the response variance. Every explanatory variable with a parameter weight exceeding the value of 0.5 will be discussed as important for the response variable (variables with parameter weights > 0.5 were part of at least some low AICc models or several high AICc models within the AICc range of two). We extracted centred and standardised estimates and standard errors for improved interpretability [45] from the summary table of the model with the lowest AICc including all important explanatory variables (parameter weight > 0.5).

**Table 1:** Correlations among explanatory variables, Pearson correlation coefficients (except for flower colour: Student’s t) and levels of significance are given with *p < 0.05, **p < 0.01 and ***p < 0.001.

	Plant size (cm)	Flower number	Flower biomass (g)	Flower colour	Fruit number	Fruit biomass (g)	Leaf number	Leaf biomass (g)
Plant size (cm)		ns	ns	8.55***	-0.48*	ns	ns	0.63**
Flower number	ns		ns	16.45***				
Flower biomass (g)	ns	ns		7.86***				
Flower colour	8.55***	16.45***	7.86***					
Fruit number	-0.48*					ns		
Fruit biomass (g)	ns				ns			
Leaf number	ns							ns
Leaf biomass (g)	0.63**						ns	
H SR leaves	0.74***						ns	0.79***
H SR fruits	0.53*				-0.62**	ns		
H SR	0.75***							
Endophagous H SR	0.80***							
Ectophagous H SR	0.66**							

Species richness = SR, herbivores = H. Number and biomass of flowers refer to plot level (N = 42 plots), while number and biomass of leaves and fruits and species richness of herbivores refer to five plant individuals per plot (N = 84 plots). Number and biomass of flowers, fruits and leaves were log-transformed, species richness of leaf herbivores was sqrt-transformed. Empty cells refer to combinations of variables which were not tested.

In order to compare the overall effects of plant size on species richness of herbivores, their natural enemies and pollinators, we calculated additional linear mixed effects models (plant species as random effect; based on the full data set, i.e. 84 plots; species richness of natural enemies with square-root-transformation) and extracted p-values for Wald tests and slopes from the summary table as well as marginal and conditional R^2^ using the lmmR2 function incorporated in the lmmfit package of R [46].

Finally, we used a principal coordinates analysis (PCoA) as classical multidimensional scaling method to analyse the community composition of plant associated arthropods and consequently its association with plant size (using the packages vegan and BiodiversityR to add species scores [37,47]). The matrix of arthropod species (abundance data of herbivores, their natural enemies and pollinators per plant species based on the full data set, i.e. 84 plots, Bray-Curtis dissimilarity) and plant species was calculated independently of plant size. Then, we fitted plant size as environmental vector onto the ordination using the envfit-function with 1000 permutations (R^2^ and p-values deriving from estimated distribution of plant size under the null-hypothesis through permutations).

## Results

Overall we recorded 13,449 herbivores of 24 species (arithmetic mean ± SE: 5.37 ± 0.28 species per five plant individuals), 1758 natural enemies of 56 species (3.13 ± 0.25 species per five plant individuals) and 3538 pollinators of 79 species (8.49 ± 0.43 species per plot) (see S2 Table for means ± SE of the different plant species; see S3 Table for a list of observed species; see S4 Table for raw data). Among the herbivores, we sampled 2.25 ± 0.18 endophagous species per five plant individuals (1.98 ± 0.20 natural enemy species of endophagous prey or hosts) and 3.80 ± 0.21 ectophagous species per five plant individuals (1.15 ± 0.13 natural enemy species of endophagous prey or hosts). 2.89 ± 0.21 herbivore species were found in and on leaves (1.01 ± 0.13 natural enemy species), 1.36 ± 0.10 herbivore species in and on fruits (1.71 ± 0.17 natural enemy species), 1.88 ± 1.36 herbivore species in and on stems (0.33 ± 0.61 natural enemy species) and 2.05 ± 1.19 herbivore species in and on flowers (0.86 ± 0.93 natural enemy species) of five plant individuals.

**Fig 1:**
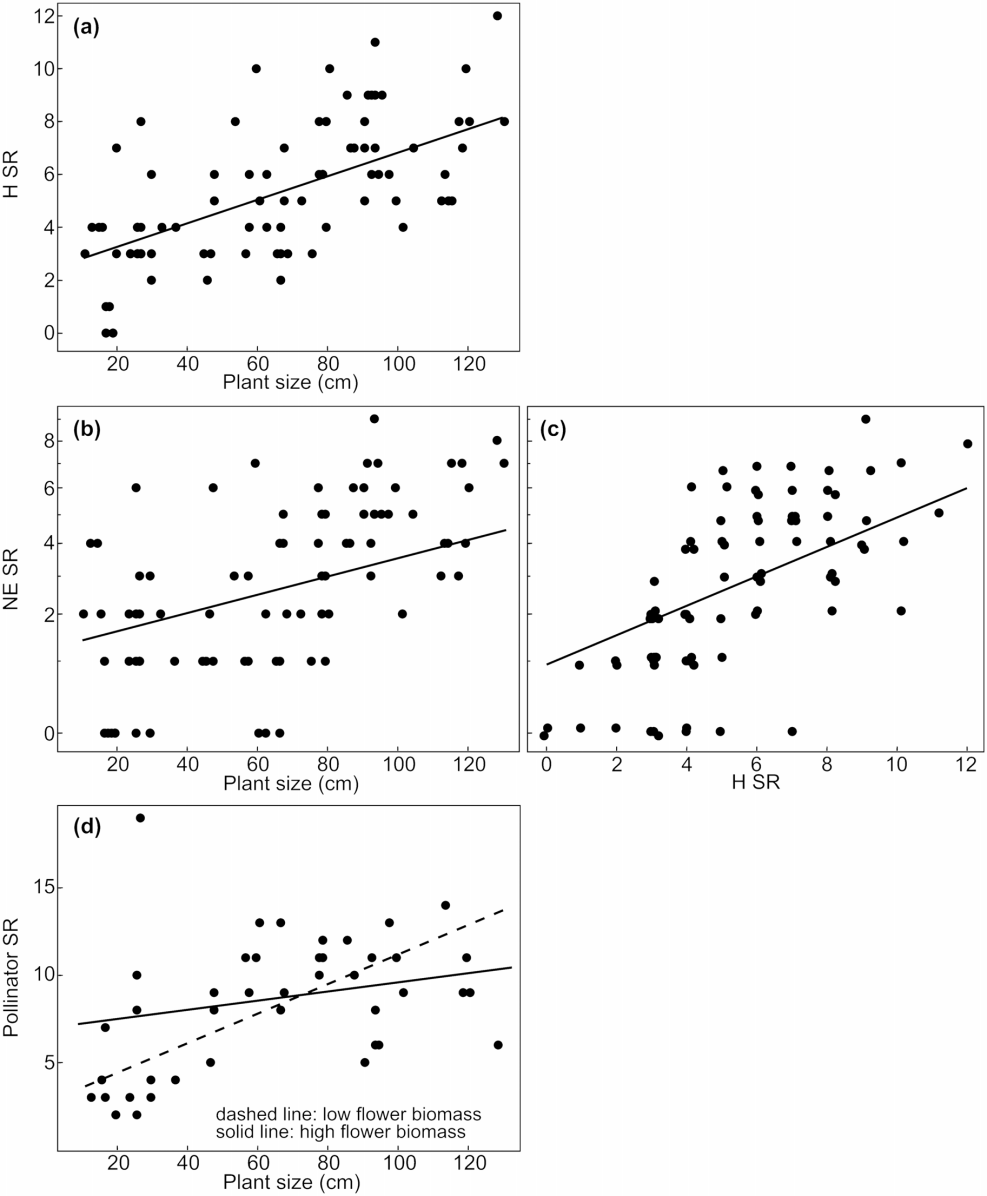
Effects of plant size and covariables on species richness of different groups of arthropods. Effects of plant size on species richness of (a) herbivores, (b) their natural enemies and (d) pollinators. Additionally, effects of important covariables representing the amount of food resource for natural enemies are shown (c). SR = species richness, H = herbivores, NE = natural enemies. Species richness of pollinators refer to plot level (N = 42 plots), while species richness of herbivores and natural enemies refer to five plant individuals per plot (N = 84 plots). Axes of variables were transformed corresponding to analyses (species richness of natural enemies: sqrt-transformation). Data points in (c) were jittered. Predictions derive from the lme-model with the lowest AICc including all explanatory variables with a parameter weight > 0.5. To visualise interactions of two continuous explanatory variables (d), we converted one of them into a categorical variable, using the medians of the upper and the lower half of the data (dashed line: low flower biomass = 13 g, solid line: high flower biomass = 39 g).

**Fig 2:**
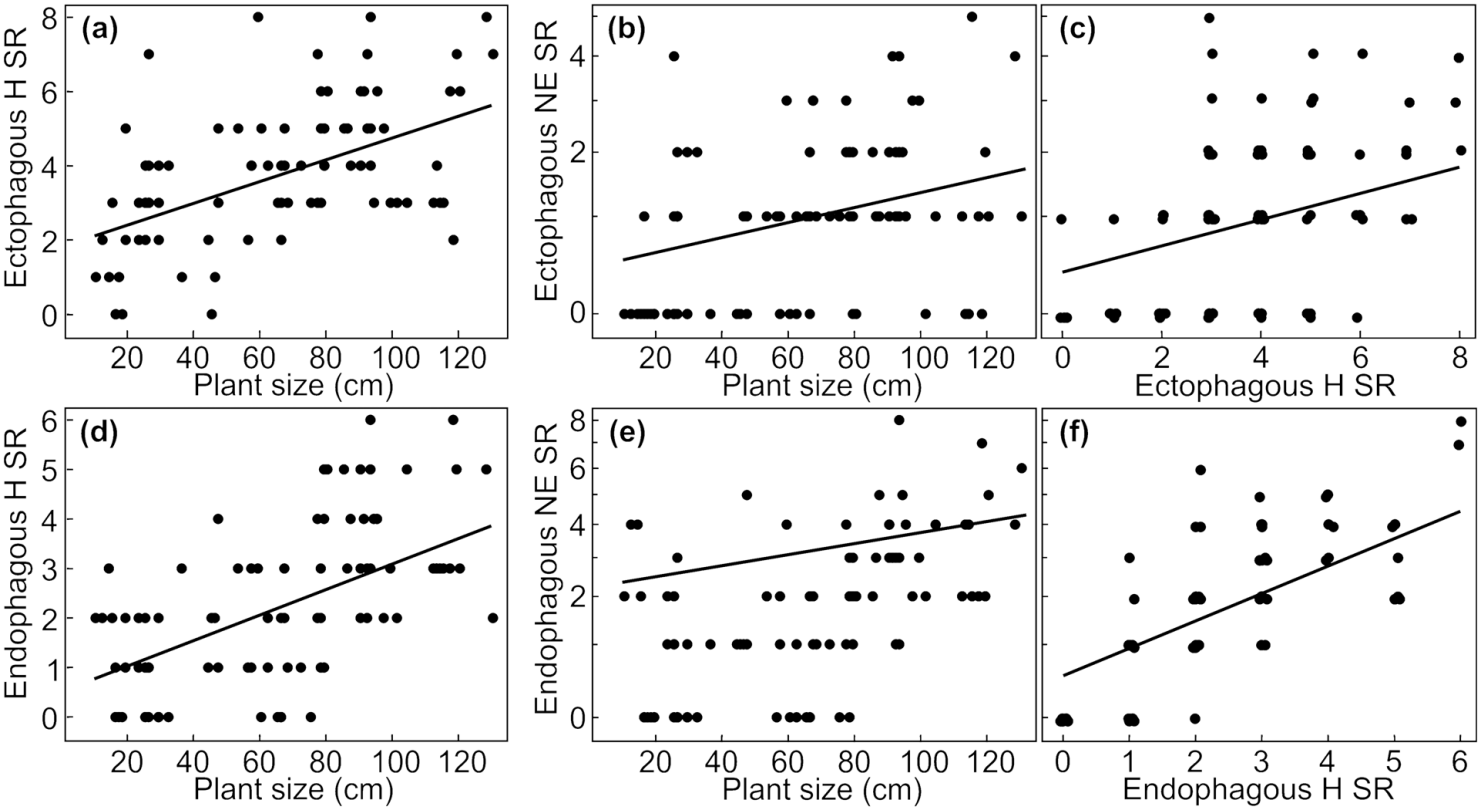
Effects of plant size and covariables on species richness of ectophagous and endophagous arthropods. Effects of plant size on species richness of (a) ectophagous and (d) endophagous herbivores and of (b, e) their respective natural enemies. Additionally, effects of important covariables representing the amount of food resource for (c, f) natural enemies are shown. SR = species richness, H = herbivores, NE = natural enemies. Species richness of herbivores and natural enemies refer to five plant individuals per plot (N = 84 plots). Axes of variables were transformed corresponding to analyses (species richness of natural enemies: sqrt-transformation). Data points in (c, f) were jittered. Predictions derive from the lme-model with the lowest AICc including all explanatory variables with a parameter weight > 0.5.

**Fig 3:**
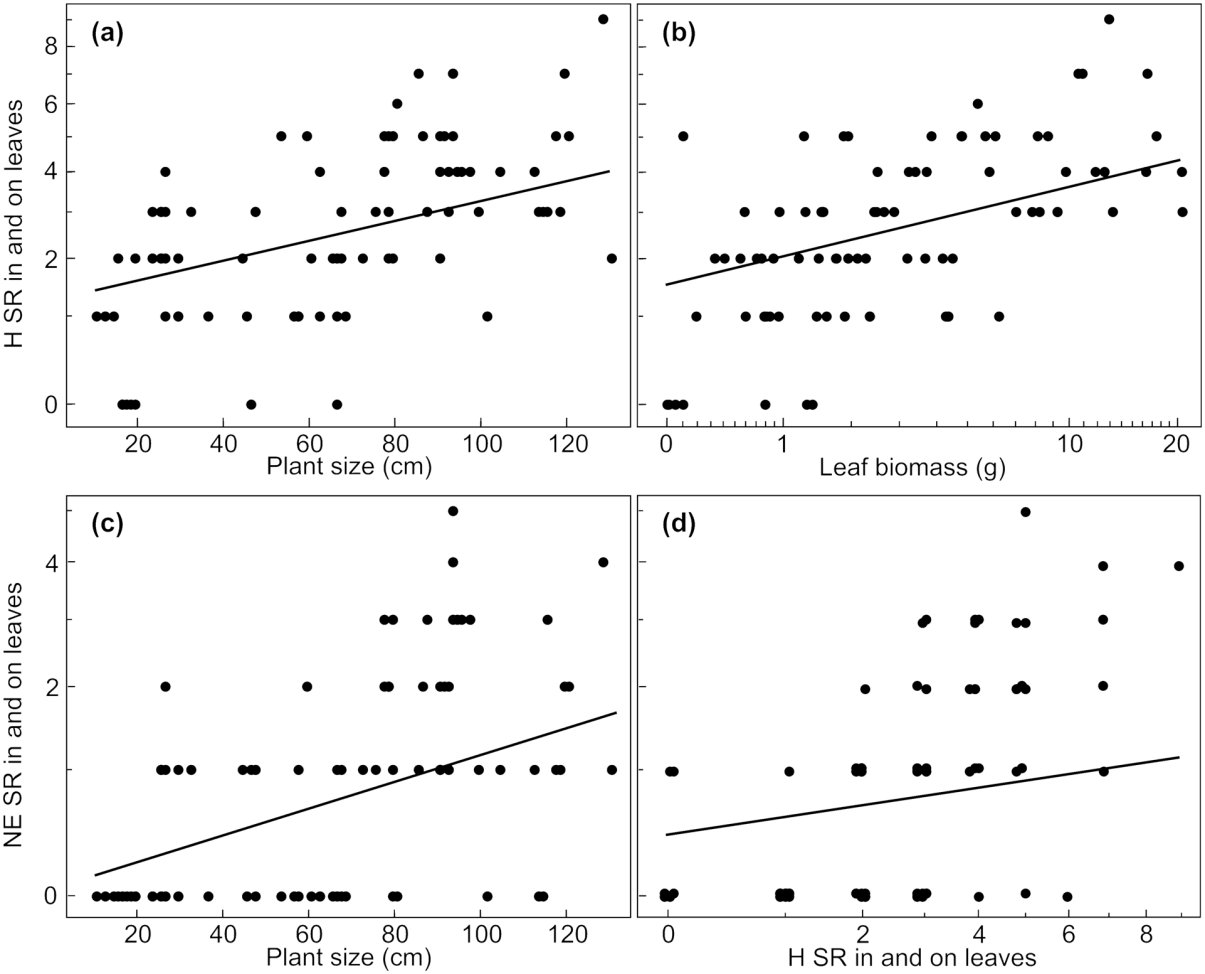
Effects of plant size and covariables on species richness of leaf associated arthropods. Effects of plant size on species richness of (a) leaf associated herbivores and (c) their natural enemies. Additionally, effects of important covariables representing the amount of food resource for (b) herbivores and (d) for their natural enemies are shown. SR = species richness, H = herbivores, NE = natural enemies. Leaf biomass as well as species richness of herbivores and natural enemies refer to five plant individuals per plot (N = 84 plots). Axes of variables were transformed corresponding to analyses (species richness of leaf associated herbivores and species richness of their natural enemies: sqrt-transformation, leaf biomass: log-transformation). Data points in (d) were jittered. Predictions derive from the lme-model with the lowest AICc including all explanatory variables with a parameter weight > 0.5.

**Fig 4:**
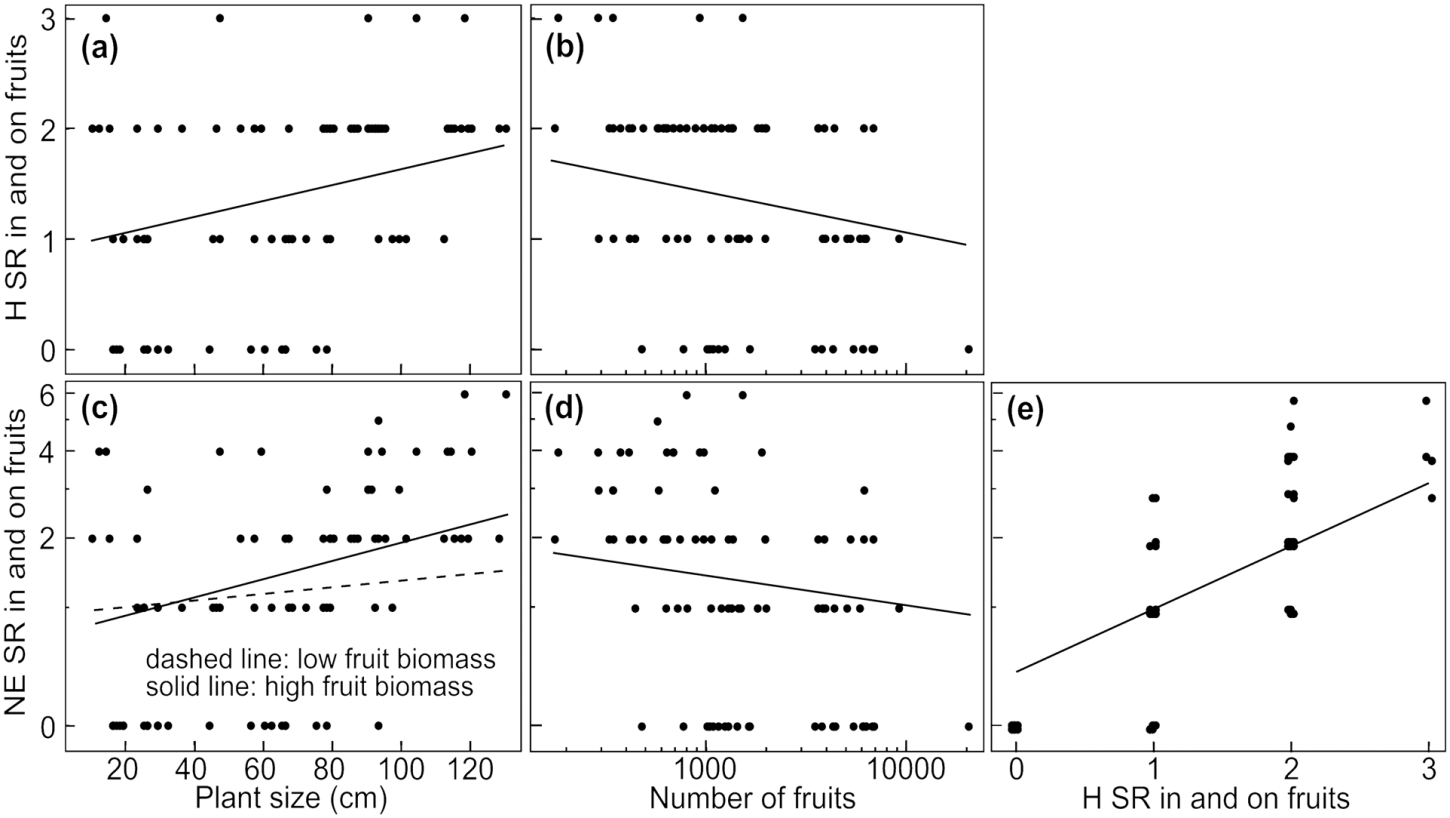
Effects of plant size and covariables on species richness of fruit associated arthropods. Effects of plant size on species richness of (a) fruit associated herbivores and (c) their natural enemies. Additionally, effects of important covariables representing the amount of food resource for (b) herbivores and for (d-e) their natural enemies are shown. SR = species richness, H = herbivores, NE = natural enemies. Number of fruits and species richness of herbivores and natural enemies refer to five plant individuals per plot (N = 84 plots). Axes of variables were transformed corresponding to analyses (species richness of natural enemies and number of fruits: log-transformation). Data points in (e) were jittered. Predictions derive from the lme-model with the lowest AICc including all explanatory variables with a parameter weight > 0.5. To visualise interactions of two continuous explanatory variables (c), we converted one of them into a categorical variable, using the medians of the upper and the lower half of the data (dashed line: low fruit biomass = 9.20 g, solid line: high fruit biomass = 39.38 g).

The results of the different models show a comprehensive influence of plant size on species richness of associated arthropods. Overall species richness of herbivores, their natural enemies and pollinators increased with increasing plant size (Fig 1a, 1b and 1d, Tables 2 and 3). This positive effect of plant size held not only for estimated herbivore species richness (ACE estimator [48]; S1 File), but also for ectophagous and endophagous herbivores and their natural enemies as well as for fruit and leave herbivores and their natural enemies (Figs 2a, 2b, 2d, 2e, 3a, 3c and 4a, 4c, Tables 2, 4 and 5).

**Table 2:** Effects of plant size and covariables on species richness (SR) of herbivores (H) and their natural enemies (NE). Species richness of herbivores and natural enemies refer to five plant individuals per plot (N = 84 plots). Species richness of natural enemies (in total and of ectophagous vs. endophagous prey/hosts) were sqrt-transformed. Parameter weights (pw) refer to a delta 2 AICc range. Explanatory variables and interactions with a parameter weight > 0.5 were considered as important for the relevant response variable and are shown in **bold**. Estimates (est) with standard errors (SE) were assessed from the summary table of the lme-model with the lowest AICc including all explanatory variables with a parameter weight > 0.5 and are centred and standardised to improve their interpretability. Empty cells refer to variables which were not involved in the relevant full model.

		Plant size (cm)	H SR	Plant size: H SR
H SR	pw	**1.000**		
	est	**1.491**		
	SE	**0.319**		
Ectophagous	pw	**1.000**		
	est	**0.986**		
	SE	**0.260**		
Endophagous	pw	**1.000**		
	est	**0.866**		
	SE	**0.211**		
NE SR	pw	**1.000**	**1.000**	0.000
	est	**0.240**	**0.292**	-
	SE	**0.076**	**0.069**	-
Ectophagous	pw	**1.000**	**1.000**	0.000
	est	**0.142**	**0.142**	-
	SE	**0.058**	**0.055**	-
Endophagous	pw	**0.760**	**1.000**	0.022
	est	**0.143**	**0.338**	-
	SE	**0.065**	**0.046**	-

**Table 3:** Effects of plant size and covariables on species richness (SR) of pollinators. Number and biomass of flowers and species richness of pollinators refer to plot level (N = 42 plots). Number and biomass of flowers were log-transformed. For further information see caption of Table 2.

		Plant size (cm)	Flower number	Flower biomass (g)	Flower colour	Plant size: flower number	Plant size: flower biomass	Plant size: flower colour	Flower number: flower biomass	Flower number: flower colour	Flower biomass: flower colour
Pollinator SR	pw	**1.000**	0.490	**1.000**	0.200	0.000	**1.000**	0.000	0.490	0.000	0.000
	est	**2.100**	-	**0.390**	-	-	**-1.506**	-	-	-	-
	SE	**0.529**	-	**0.591**	-	-	**0.522**	-	-	-	-

**Table 4:** Effects of plant size and covariables on species richness (SR) of leaf associated herbivores (H) and their natural enemies (NE). Number and biomass of leaves and species richness of herbivores and natural enemies refer to five plant individuals per plot (N = 84 plots). Number and biomass of leaves were log-transformed, species richness of leaf associated herbivores and of their natural enemies were sqrt-transformed. For further information see caption of Table 2.

		Plant size (cm)	Leaf number	Leaf biomass (g)	Plant size: leaf number	Plant size: leaf biomass	Leaf number: leaf biomass	H SR	H SR: Plant size	H SR: number leaves	H SR: biomass leaves
H SR leaves	pw	**1.000**	0.000	**1.000**	0.000	0.000	0.000				
	est	**0.212**	-	**0.210**	-	-	-				
	SE	**0.068**	-	**0.061**	-	-	-				
NE SR leaves	pw	**1.000**	0.000	0.340	0.000	0.000	0.000	**1.000**	0.000	0.000	0.000
	est	**0.202**	-	-	-	-	-	**0.079**	-	-	-
	SE	**0.058**	-	-	-	-	-	**0.040**	-	-	-

**Table 5:** Effects of plant size and covariables on species richness (SR) of fruit associated herbivores (H) and their natural enemies (NE). Number and biomass of fruits and species richness of herbivores and natural enemies refer to five plant individuals per plot (N = 84 plots). Number and biomass of fruits and species richness of fruit associated natural enemies were log-transformed. For further information see caption of Table 2.

		Plant size (cm)	Fruit number	Fruit biomass (g)	Plant size: fruit number	Plant size: fruit biomass	Fruit number: fruit biomass	H SR	H SR: Plant size	H SR: number fruits	H SR: biomass fruits
H SR fruits	pw	**0.750**	**0.750**	0.240	0.130	0.000	0.000				
	est	**0.242**	**-0.165**	-	-	-	-				
	SE	**0.130**	**0.112**	-	-	-	-				
NE SR fruits	pw	**1.000**	**0.610**	**0.720**	0.090	**0.530**	0.000	**1.000**	0.090	0.000	0.000
	est	**0.126**	**-0.079**	**0.070**	-	**0.069**	-	**0.324**	-	-	-
	SE	**0.055**	**0.046**	**0.042**	-	**0.038**	-	**0.037**	-	-	-

In addition to the direct effect of plant size, the different covariables referring to resource quantity had an effect on species richness of the different organism groups. The positive effect of herbivore species richness on observed species richness of their natural enemies was likewise true for estimated natural enemy species richness (ACE estimator [48]; S1 File), for natural enemies of endophagous, ectophagous, fruit and leaf associated herbivores (Figs 1c, 2c, 2f, 3d and 4e; Tables 2, 4 and 5). Species richness of leaf associated herbivores was positively affected by leaf biomass but not by leaf number (Fig 3b, Table 4), while leaf biomass and number had no effect on natural enemy species richness of leaf associated herbivores (Table 4). Fruit number had a negative effect on species richness of fruit associated herbivores and their natural enemies (Fig 4b and 4d, Table 5). Species richness of fruit associated herbivores was not affected by fruit biomass, while the positive effect of plant size on their natural enemies was enhanced by increasing fruit biomass (Fig 4c, Table 5). The positive effect of plant size on pollinator species richness was enhanced by flower biomass (Fig 1d, Table 3), while flower number and colour (yellow/white) had no effect on species richness of flower visiting pollinators (Table 3).

Comparing the overall effects of plant size on species richness of herbivores, their natural enemies and pollinators (all p-values < 0.001; slopes for herbivores: 0.047, their natural enemies: 0.013, pollinators: 0.063), we found a higher marginal R^2^ in case of herbivores and their natural enemies compared to pollinators (marginal R^2^ of herbivores: 0.414, their natural enemies: 0.403, pollinators: 0.222; conditional R^2^ of herbivores: 0.685, their natural enemies: 0.627, pollinators: 0.699).

The effects of plant size on species richness of different groups of associated organisms were also reflected by community composition analysis using PCoA (association between ordination and plant size: R^2^ = 0.630, p < 0.001). The communities associated with large and small plant species, respectively, were diverging along the plant size gradient (S2 Fig). Many different species of pollinators, herbivores and their natural enemies made up the community of large plants, among them highly abundant species such as the honeybee *Apis mellifera* L., the pollen beetle *Meligethes aeneus* Fabricius and *Scaptomyza flava* Fallen. The communities on small plants comprised species such as the small wild bee *Andrena* cf. *minutuloides* Perkins and the spider *Metellina segmentata* (Clerck), which did not occur in high abundance in our study.

## Discussion

In broad support of our first hypothesis, we observed a positive effect of plant size on species richness of plant associated herbivores (overall, ectophagous, endophagous, leaf and fruit associated), their natural enemies and pollinators. The likelihood of being found and colonised by associated arthropod species increases with increasing plant size due to factors such as increasing visual and chemical conspicuousness and attractiveness (e.g. apparency, resource diversity, plant vigour and growth-defense trade-off hypotheses [6,9–11]). Increases in species richness with increasing plant size were shown by several studies, mostly focussing on herbivorous organism groups [12–14]. In our study, the positive effect of plant size on herbivore species richness held for ectophagous, endophagous, leaf and fruit associated herbivores. The response strength was similar for ectophagous and endophagous herbivores, indicating similar mechanisms of host plant choice. Accordingly, some studies found increasing herbivore species richness in ectophagous herbivore species richness [12,14,49], while other studies showed a positive effect of plant size on endophagous insect larvae with increasing plant size [13,16]. Endophagous larvae have to utilise the host plant their egg-laying mother chose and are not able to switch to another plant. However, adults of endophagous larvae may prefer large plants because they are conspicuous and attractive microhabitats for their offspring, leading to a positive effect of plant size on species richness in endophagous species as well. The closer inspection of herbivores on single plant organs (leaves and fruits) provided the opportunity to disentangle effects of plant size and resource availability, with the result that the positive effect of plant size on species richness of herbivores persisted. Increasing plant size may lead to increased visual and chemical conspicuousness of single plant organs, particularly of those positioned at the top of a plant. Given that large plants offer a wider range of resources and niches, larger microhabitat area, higher plant vigour or lower levels of defensive compounds than small plants [9–11], large plants can be highly attractive even to herbivores of organs which are hidden inside the vegetation. In accordance with our findings, leaf associated caterpillar densities on *Calluna vulgaris* were reported to increase with increasing intraspecific plant height while controlling for resource availability [12].

Moreover, our results showed positive effects of leaf biomass on species richness of leaf associated herbivores. High leaf biomass indicates high food amount followed by lowered competition among herbivores and can decrease predation risk as dense and complex foliage structure can provide refuge from natural enemies [9,50]. Surprisingly, species richness of fruit herbivores (exclusively endophagous herbivores) was negatively related to fruit number, which in turn was negatively correlated with fruit size (not incorporated in the model). It is likely that the negative effect of fruit number represented a positive effect of fruit size, as large fruits are highly conspicuous (visually and chemically via e.g. high emission of volatiles) and offer high microhabitat area and food resources to mining herbivores.

Natural enemy species are often specialised on specific prey or hosts. Therefore high species richness of herbivorous insects promotes species richness of natural enemies [20,21], which is supported by our findings of a positive effect of herbivore species richness on species richness of their natural enemies throughout all analyses. Predators and parasitoids can locate their prey and hosts via direct, mostly chemical, cues and in an indirect way (e.g. by herbivore-induced plant volatiles and by choosing the habitat of their prey or host), often in a combination of both [22,23]. Despite simultaneous consideration of prey and host species richness, natural enemy species richness increased with increasing plant size throughout all analyses. This is in accordance to Hawkins et al. [51], showing a positive effect of host food plant size on species richness of parasitoids of ectophagous and endophagous hosts. These results suggest that large plants are attractive foraging sites for natural enemies since they offer conspicuous and attractive microhabitats for their prey or hosts. Species richness of natural enemies of ectophagous herbivores (mainly composed of predators) was promoted by herbivore species richness in a similar degree as by plant size, while species richness of natural enemies of endophagous herbivores (composed of parasitoids) was more influenced by their hosts’ species richness than by plant size. Parasitoids are usually highly specialised on single herbivore taxa and react sensitively to kairomones of their hosts and on herbivore-induced plant volatiles [8,23,52], while predators use a wider range of prey species [16,53]. The closer inspection of natural enemies of leaf and fruit associated herbivores again showed a similarity in effects of plant characteristics on species richness of herbivores and their natural enemies. Besides a positive effect of leaf herbivore species richness, species richness of their natural enemies increased with increasing plant size, thereby following their prey and hosts in the selection of conspicuous and attractive large plants. Likewise following the patterns of their host organisms, also species richness of fruit associated natural enemies increased with increasing plant size and decreased with increasing fruit number besides a positive effect of fruit herbivore species richness. Consecutively, it seems to be worthwhile for natural enemies to follow herbivores in the selection of large plants and large fruits (fruit number was negatively correlated with fruit size, which was not incorporated in the model). Here the positive effect of plant size on their parasitoids was reinforced by high fruit biomass, indicating numerous and large fruits. This enhancement of conspicuousness for natural enemies of fruit associated herbivores (exclusively parasitoids) by many and large fruits at the plant’s top can be of visual and particularly of olfactory nature, as fruits of many plant species contain oils and semiochemicals attracting natural enemies, notably parasitoids [52,54].

Not only herbivores and their natural enemies, but also pollinators have been shown to be affected by plant size when intraspecific variability is high [27–29, but see 43]. Petals of flowers visually and chemically attract pollinating animals and their attractiveness should be enhanced by a location superior to other flowers [25]. Plant size therefore can play an important role in plants with inflorescences located at the very top of the plant, like in the tested Brassicaceae, where flowers of large plants were more exposed than those of small plants. The generally positive effect of plant size on pollinator species richness was weaker on plants with high flower biomass than on plants with low flower biomass. High flower biomass combined the positive effects of flower number and flower size. Numerous flowers not only support the flowers’ visual and chemical conspicuousness to pollinators, but additionally offer a large amount of pollen and nectar with low foraging distances between flowers and a low competition among pollinators [55,56]. Large flowers are not only highly conspicuous but also attractive to pollinators as they often signal high production of nectar and pollen [25 and studies cited therein,^26^]. High flower biomass weakened the positive effect of plant size on pollinator species richness, suggesting that the visual and chemical conspicuousness and attractiveness of plants with high flower biomass was already high and was only weakly increased by a high position of the flowers due to high plant size. Although single species of pollinators can have flower colour preferences for e.g. yellow [57], flower colour (yellow vs. white without considering other characteristics such as wavelengths or UV signals) turned out to have no effect on pollinator species richness in this study.

Comparing the overall effect of plant size on species richness of the different organism groups, we found a higher marginal R^2^ in case of herbivores and their natural enemies compared to pollinators. This comparatively lower proportion of explained variance (R^2^) regarding pollinator species richness is in line with the results of the linear models involving covariables, which emphasised the importance of flower characteristics for pollinator species richness besides plant size.

Finally, the effect of plant size on the different organism groups consequently led to an effect on the community composition. Communities of large plants were formed by different species in comparison to those of small plants. The domination of communities of large plants by numerous, particularly highly abundant species such as honey bees and pollen beetles can be ascribed to the high conspicuousness and attractiveness of large plants for arthropod individuals. Since these highly abundant species are known to influence the seed output of the plant [58,59], differences in plant size may thereby lead to important functional consequences for plant performance.

## Conclusions

**Fig 5:**
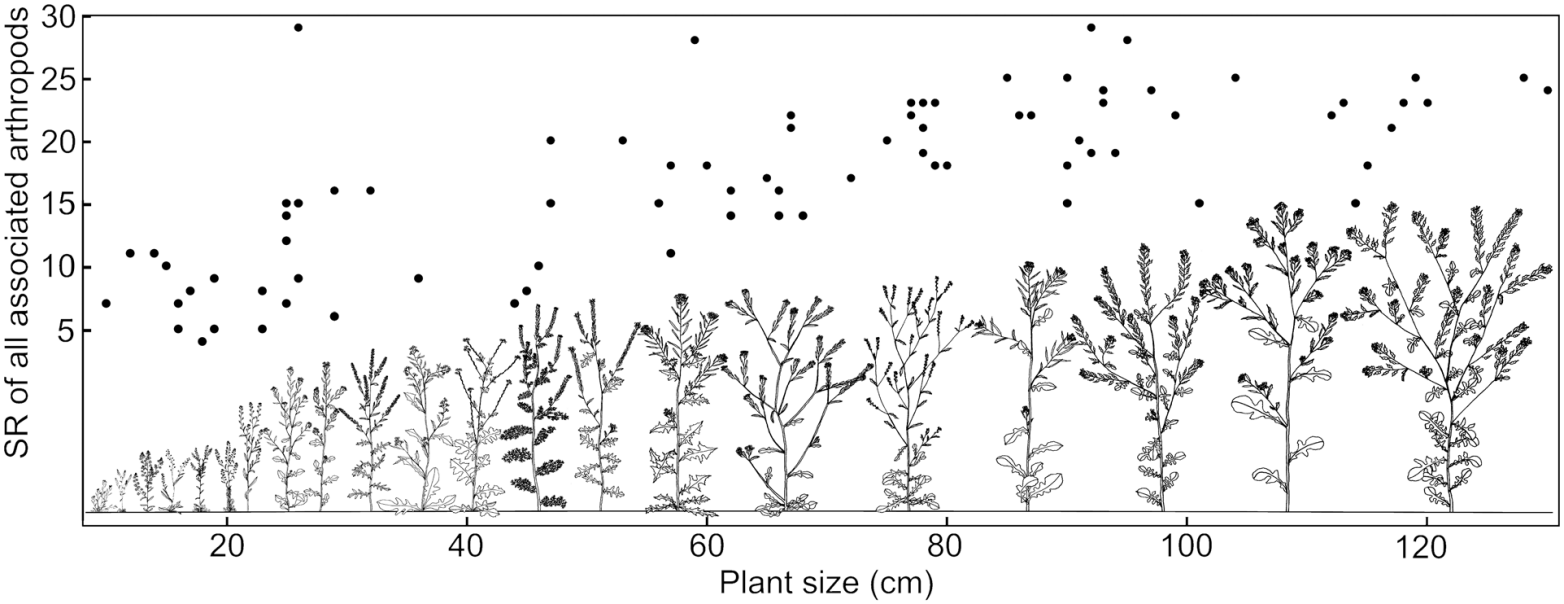
Effects of plant size on total species richness of arthropods. The observed total species richness of arthropods was calculated by summing up observed species richness of herbivores and their natural enemies per five plant individuals of every plot and observed species richness of pollinators per plot (N = 84 plots; on average eight species associated with plants of heights between ten and 20 cm and 24 species associated with plants of heights between 120 and 130 cm). SR = species richness. Size of the different plant species of this study is illustrated relative to one another but independent of the x-axis by modified drawings from Schlinkert [60].

Our detailed results of insect species richness along an interspecific plant size gradient in a standardised common garden experiment exhibit a strong pattern: large plants harbour more arthropod species associated with all aboveground plant organs than small plants, including herbivores (whether they are endophagous or ectophagous, associated with leaves or fruits), their natural enemies and pollinators. An overall increase in plant size from 10 to 130 cm height led to 2.7 times higher predicted total species richness from nine to 24 associated arthropods (five vs. 13 pollinator species per plot, three vs. eight herbivore species and one vs. three natural enemy species per five plant individuals; Fig 5). This positive effect of plant size on species richness prevailed even under simultaneous consideration of resource availability for the different functional groups (i.e. organ biomass for herbivores, herbivore species richness for natural enemies and number of flowers for pollinators). In general, plant size was shown to be a comprehensive driver of species richness of the arthropod community. These findings are highly relevant as they result from a broad gradient in plant size across 21 species of Brassicaceae in a common garden experiment without confounding influences of landscape or habitat. They can be expected to apply also for other plant families that exhibit a large size gradient. However, further plant characteristics may also contribute to the explanation in other families, e.g. when also perennials (not only annuals) or woody (not only herbaceous) plants are involved. Plant size should be better acknowledged in studies focusing on diversity of arthropod communities associated with plants and factors driving the structure of these communities. On the other hand, effects of plant size on associated arthropods can have ecological consequences for individual plants, since plant fitness might be affected by size effects on mutualistic and antagonistic interaction partners.

## Supporting Information

S1 Fig Photo of the common garden experiment. (PDF)

S2 Fig Community composition ordination (PCoA). (PDF)

S1 File Abundance-based coverage estimators. (PDF)

S1 Table Correlations between organ size and other plant characteristics. (PDF)

S2 Table Means and standard errors per plant species of all tested parameters. (PDF)

S3 Table Species list. (PDF)

S4 Table Raw data. (PDF)

## References

[1] Peters RH. The ecological implications of body size. Cambridge: Cambridge University Press; 1983 10.1017/CBO9780511608551

[2] Brown JH, Maurer BA. Body size, ecological dominance and Cope’s rule. Nature. 1986;324: 248–250. Available: http://www.nature.com/nature/journal/v324/n6094/abs/324248a0.html

[3] Hemptinne J-L, Magro A, Evans EW, Dixon AFG. Body size and the rate of spread of invasive ladybird beetles in North America. Biol Invasions. 2012;14: 595–605. 10.1007/s10530-011-0101-0

[4] Blanckenhorn WU. The evolution of body size: what keeps organisms small? Q Rev Biol. 2000;75: 385–407. 10.1086/393620 11125698

[5] Remmel T, Tammaru T. Size-dependent predation risk in tree-feeding insects with different colouration strategies: a field experiment. J Anim Ecol. 2009;78: 973–980. 10.1111/j.1365-2656.2009.01566.x 19493131

[6] Feeny P. Plant apparency and chemical defense In: Wallace JW, Mansell RL, editors. Biochemical interaction between plants and insects. New York: Springer US; 1976 pp. 1–40. 10.1007/978-1-4684-2646-5_1

[7] Bruce TJA, Wadhams LJ, Woodcock CM. Insect host location: a volatile situation. Trends Plant Sci. 2005;10: 269–274. 10.1016/j.tplants.2005.04.003 15949760

[8] McCormick AC, Unsicker SB, Gershenzon J. The specificity of herbivore-induced plant volatiles in attracting herbivore enemies. Trends Plant Sci. 2012;17: 303–310. 10.1016/j.tplants.2012.03.012 22503606

[9] Lawton JH. Plant architecture and the diversity of phytophagous insects. Annu Rev Entomol. 1983;28: 23–39. Available: http://www.annualreviews.org/doi/pdf/10.1146/annurev.en.28.010183.000323

[10] Price PW. The plant vigor hypothesis and herbivore attack. Oikos. 1991;62: 244–251. Available: http://www.jstor.org/stable/3545270

[11] Herms D, Mattson W. The dilemma of plants: to grow or defend. Q Rev Biol. 1992;67: 283–335. 10.1086/417659

[12] Haysom KA, Coulson JC. The Lepidoptera fauna associated with *Calluna vulgaris*: effects of plant architecture on abundance and diversity. Ecol Entomol. 1998;23: 377–385. 10.1046/j.1365-2311.1998.00152.x

[13] Lawton JH, Price PW. Species richness of parasites on hosts: agromyzid flies on the British Umbelliferae. J Anim Ecol. 1979;48: 619–637. Available: http://www.jstor.org/stable/10.2307/4183

[14] Neuvonen S, Niemelä P. Species richness of Macrolepidoptera on Finnish deciduous trees and shrubs. Oecologia. 1981;51: 364–370. Available: http://link.springer.com/article/10.1007/BF00540907 28310021 10.1007/BF00540907

[15] Espírito-Santo MM, Neves FDS, Andrade-Neto FR, Fernandes GW. Plant architecture and meristem dynamics as the mechanisms determining the diversity of gall-inducing insects. Oecologia. 2007;153: 353–364. 10.1007/s00442-007-0737-8 17453251

[16] Tscharntke T, Greiler H-J. Insect communities, grasses, and grasslands. Annu Rev Entomol. 1995;40: 535–558. Available: http://www.annualreviews.org/doi/pdf/10.1146/annurev.en.40.010195.002535

[17] Strong DR, Lawton JH, Southwood TRE. Insects on plants: community patterns and mechanisms. Oxford: Blackwell Scientific Publications; 1984.

[18] Araujo APA, D’arc de Paula J, Carneiro MAA, Schoereder JH. Effects of host plant architecture on colonization by galling insects. Austral Ecol. 2006;31: 343–348. 10.1111/j.1442-9993.2006.01563.x

[19] Reudler Talsma JH, Biere A, Harvey JA, van Nouhuys S. Oviposition cues for a specialist butterfly-plant chemistry and size. J Chem Ecol. 2008;34: 1202–1212. 10.1007/s10886-008-9519-y 18612691 PMC2518948

[20] Hunter MD, Price PW. Playing chutes and ladders: heterogeneity and the relative roles of bottom-up and top-down forces in natural communities. Ecology. 1992;73: 724–732. Available: http://www.esf.edu/efb/parry/Insect Ecology Reading/HunterPrice1992.pdf

[21] Knops JMH, Tilman D, Haddad NM, Naeem S, Mitchell CE, Haarstad J, et al Effects of plant species richness on invasion dynamics, disease outbreaks, insect abundances and diversity. Ecol Lett. 1999;2: 286–293. Available: http://onlinelibrary.wiley.com/doi/10.1046/j.1461-0248.1999.00083.x/full 10.1046/j.1461-0248.1999.00083.x 33810630

[22] Hodek I. Habitat and food specificity in aphidophagous predators. Biocontrol Sci Technol. 1993;3: 91–100. Available: http://medcontent.metapress.com/index/A65RM03P4874243N.pdf

[23] Williams IH, Cook SM. Crop location by oilseed rape pests and host location by their parasitoids In: Williams IH, editor. Biocontrol-based integrated management of oilseed rape pests. Dordrecht: Springer Netherlands; 2010 pp. 215–244. 10.1007/978-90-481-3983-5_7

[24] Hawkins BA, Lawton JH. Species richness for parasitoids of British phytophagous insects. Nature. 1987;326: 788–790. Available: http://www.nature.com/nature/journal/v326/n6115/abs/326788a0.html

[25] Cohen D, Shmida A. The evolution of flower display and reward. Evol Biol. 1993;27: 197–243. 10.1007/978-1-4615-2878-4_6

[26] Hegland SJ, Totland Ø. Relationships between species’ floral traits and pollinator visitation in a temperate grassland. Oecologia. 2005;145: 586–594. 10.1007/s00442-005-0165-6 16028095

[27] Leong JM, Thorp RW. Colour-coded sampling: the pan trap colour preferences of oligolectic and nonoligolectic bees associated with a vernal pool plant. Ecol Entomol. 1999;24: 329–335. 10.1046/j.1365-2311.1999.00196.x

[28] Raguso R. Wake up and smell the roses: the ecology and evolution of floral scent. Annu Rev Ecol Evol Syst. 2008;39: 549–569. Available: http://www.jstor.org/stable/30245176

[29] Gómez JM. Herbivory reduces the strength of pollinator-mediated selection in the Mediterranean herb *Erysimum mediohispanicum*: consequences for plant specialization. Am Nat. 2003;162: 242–256. 10.1086/376574 12858267

[30] Donnelly SE, Lortie CJ, Aarssen LW. Pollination in *Verbascum thapsus* (Scrophulariaceae): the advantage of being tall. Am J Bot. 1998;85: 1618–1625. Available: http://www.amjbot.org/content/85/11/1618.short 21680322

[31] Geber MA. The relationship of plant size to self-pollination in *Mertensia ciliata* . Ecology. 1985;66: 762–772.

[32] Southwood TRE. Tactics, strategies and templets. Oikos. 1988;52: 3–18.

[33] Buchanan AL, Underwood N. Attracting pollinators and avoiding herbivores: insects influence plant traits within and across years. Oecologia. 2013;173: 473–82. 10.1007/s00442-013-2629-4 23456243

[34] Klotz S, Kühn I, Durka W. BIOLFLOR—Eine Datenbank zu biologisch-ökologischen Merkmalen der Gefäßpflanzen in Deutschland (BIOLFLOR database—search and information system on vascular plants in Germany) Schriftenreihe für Vegetationskunde. Bonn: Bundesamt für Naturschutz; 2002.

[35] Westoby M. The self-thinning rule. Adv Ecol Res. 1984; 167–226.

[36] Byrne DN, Bellows TSJ. Whitefly biology. Annu Rev Entomol. 1991;36: 431–457. Available: http://www.annualreviews.org/doi/pdf/10.1146/annurev.en.36.010191.002243

[37] Oksanen J, Blanchet FG, Kindt R, Legendre P, Minchin PR, O’Hara RB, et al. vegan: community ecology package. R package version 2.2–1. http://CRAN.R-project.org/package=vegan. 2015.

[38] Pinheiro J, Bates D, DebRoy S, Sarkar D, R Development Core Team. nlme: linear and nonlinear mixed effects models. R package version 3.1–120. http://CRAN.R-project.org/package=nlme%3E. 2015.

[39] R Development Core Team. R: a language and environment for statistical computing. R Foundation for Statistical Computing, Vienna, Austria. URL http://www.R-project.org/. 2015.

[40] Heiberger RM. HH: statistical analysis and data display: Heiberger and Holland. R package version 3.1–15. http://CRAN.R-project.org/package=HH. 2015.

[41] Zuur AF, Ieno EN, Elphick CS. A protocol for data exploration to avoid common statistical problems. Methods Ecol Evol. 2010;1: 3–14. 10.1111/j.2041-210X.2009.00001.x

[42] Barton K. MuMIn: multi-model inference. R package version 1.13.4. http://CRAN.R-project.org/package=MuMIn. 2015.

[43] Burnham KP, Anderson DR. Model selection and multi-model inference: a practical information-theoretic approach [Internet]. 2nd ed New York: Springer New York; 2002 Available: http://amstat.tandfonline.com/doi/pdf/10.1198/tech.2003.s147

[44] Grueber CE, Nakagawa S, Laws RJ, Jamieson IG. Multimodel inference in ecology and evolution: challenges and solutions. J Evol Biol. 2011;24: 699–711. 10.1111/j.1420-9101.2010.02210.x 21272107

[45] Schielzeth H. Simple means to improve the interpretability of regression coefficients. Methods Ecol Evol. 2010;1: 103–113. 10.1111/j.2041-210X.2010.00012.x

[46] Maj A. lmmfit: Goodness-of-fit-measures for linear mixed models with one-level-grouping. R package version 1.0. http://CRAN.R-project.org/package=lmmfit. 2011.

[47] Kindt, R. & Coe R. BiodiversityR package: Tree diversity analysis. A manual and software for common statistical methods for ecological and biodiversity studies. World Agroforestry Centre (ICRAF), Nairobi. ISBN 92-9059-179-X. 2005.

[48] Chao A, Ma M-C, Yang MCK. Stopping rules and estimation for recapture debugging and unequal failure rates. Biometrika. 1993;80: 193–201.

[49] Bach CE. Host plant growth form and diversity: effects on abundance and feeding preference of a specialist herbivore, *Acalymma vittata* (Coleoptera: Chrysomelidae). Oecologia. 1981;50: 370–375. Available: http://link.springer.com/article/10.1007/BF00344978 28309056 10.1007/BF00344978

[50] Riihimaeki J, Vehvilaeinen H, Kaitaniemi P, Koricheva J. Host tree architecture mediates the effect of predators on herbivore survival. Ecol Entomol. 2006;31: 227–235. Available: http://onlinelibrary.wiley.com/doi/10.1111/j.1365-2311.2006.00784.x/full

[51] Hawkins BA, Askew RR, Shaw MR. Influences of host feeding-niche and foodplant type on generalist and specialist parasitoids. Ecol Entomol. 1990;15: 275–280.

[52] Rutledge CE. A survey of identified kairomones and synomones used by insect parasitoids to locate and accept their hosts. Chemoecology. 1996;7: 121–131. 10.1007/BF01245964

[53] Ulber B, Williams IH, Klukowski Z, Luik A, Nilsson C. Parasitoids of oilseed rape pests in Europe: key species for conservation biocontrol In: Williams IH, editor. Biocontrol-based integrated management of oilseed rape pests. Dordrecht: Springer Netherlands; 2010 pp. 45–76.

[54] Murchie AK, Smart LE, Williams IH. Responses of *Dasineura brassicae* and its parasitoids *Platygaster subuliformis* and *Omphale clypealis* to field traps baited with organic isothiocyanates. J Chem Ecol. 1997;23: 917–926. Available: http://link.springer.com/article/10.1023/B:JOEC.0000006380.59526.bd

[55] Klinkhamer PGL, de Jong TJ, de Bruyn G-J. Plant size and pollinator visitation in *Cynoglossum officinale* . Oikos. 1989;54: 201–204.

[56] Ohashi K, Yahara T. Visit larger displays but probe proportionally fewer flowers: counterintuitive behaviour of nectar-collecting bumble bees achieves an ideal free distribution. Funct Ecol. 2002;16: 492–503. 10.1046/j.1365-2435.2002.00644.x

[57] Kay QON. Preferential pollination of yellow-flowered morphs of *Raphanus raphanistrum* by *Pieris* and *Eristalis* spp. Nature. Nature Publishing Group; 1976;261: 230–232. 10.1038/261230a0

[58] Williams IH. The major insect pests of oilseed rape in Europe and their management: an overview In: Williams IH, editor. Biocontrol-based integrated management of oilseed rape pests. Dordrecht: Springer Netherlands; 2010 pp. 1–44. 10.1007/978-90-481-3983-5

[59] Sabbahi R, De Oliveira D, Marceau J. Influence of honey bee (Hymenoptera: Apidae) density on the production of canola (Crucifera: Brassicacae). J Econ Entomol. 2005;98: 367–372. 10.1603/0022-0493-98.2.367 15889725

[60] Schlinkert H. Plant size gradient in Brassicaceae. figshare, http://figshare.com; 2014; 10.6084/m9.figshare.1246843

