# Supplementary material for: Plant Size as Determinant of Species Richness of Herbivores, Natural Enemies and Pollinators across 21 Brassicaceae Species
Source: PLoS One. 2015 Aug 20;10(8):e0135928. doi: 10.1371/journal.pone.0135928 (PMC4546192; doi:10.1371/journal.pone.0135928)
Supplement: S1 Fig — (PDF) [file pone.0135928.s001.pdf]

Supporting Information S1 Fig.: Photo of the common garden experiment.

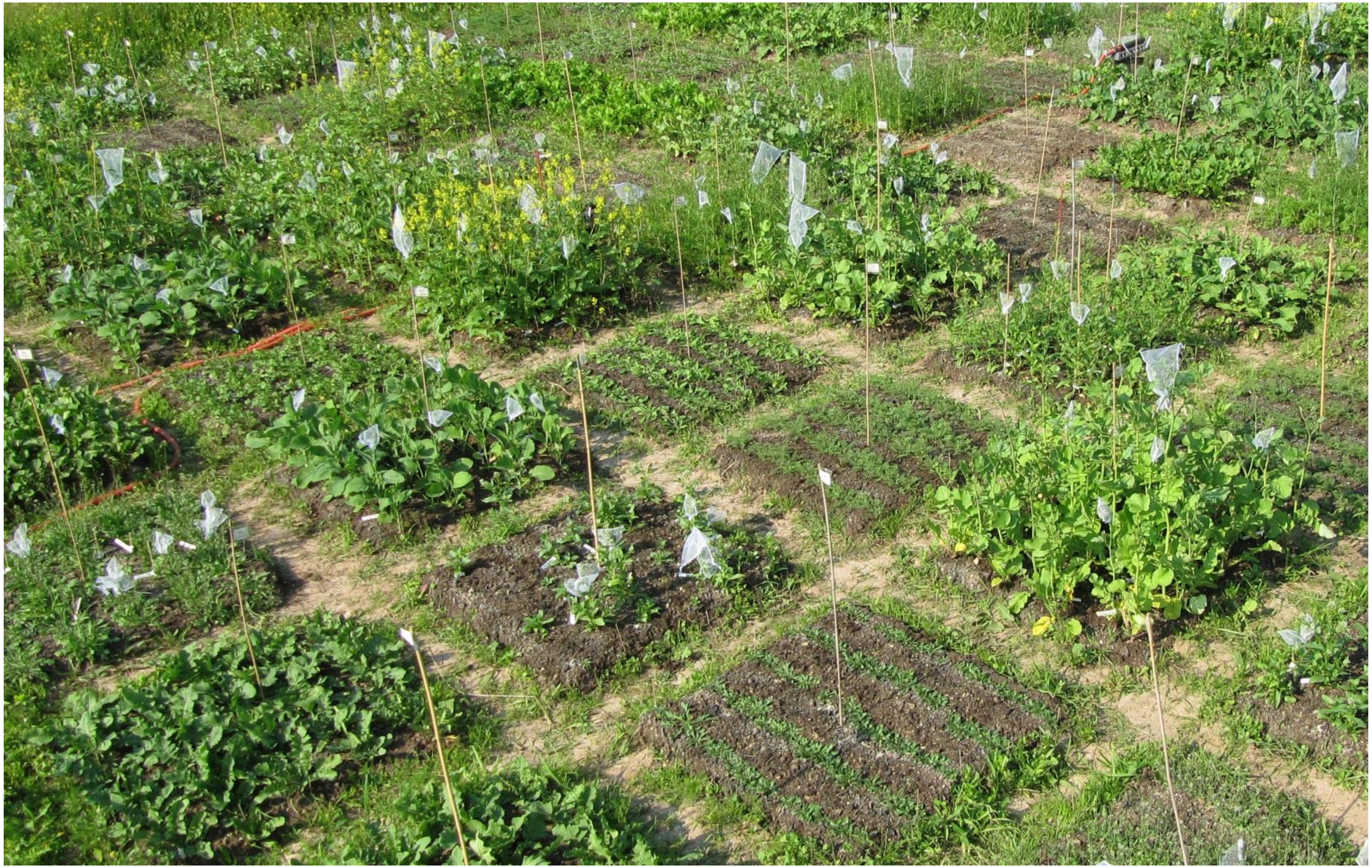

Fig. S1. Four 1 m² plots per plant species were arranged in a randomised design. We excluded plant species from the data set that could not be (1) managed to reach a plant cover of approximately 100 % of the plot or (2) brought to full flowering between mid-June and mid-July 2010 in order to avoid phenological differences in the local insect community of the study area. Plant individuals with pollinator exclusion bags were part of another experiment and were excluded from insect and plant trait samplings.
