# Supplementary material for: Plant Size as Determinant of Species Richness of Herbivores, Natural Enemies and Pollinators across 21 Brassicaceae Species
Source: PLoS One. 2015 Aug 20;10(8):e0135928. doi: 10.1371/journal.pone.0135928 (PMC4546192; doi:10.1371/journal.pone.0135928)
Supplement: S2 Fig — (PDF) [file pone.0135928.s002.pdf]

Supporting Information S2 Fig.: Community composition ordination (PCoA).

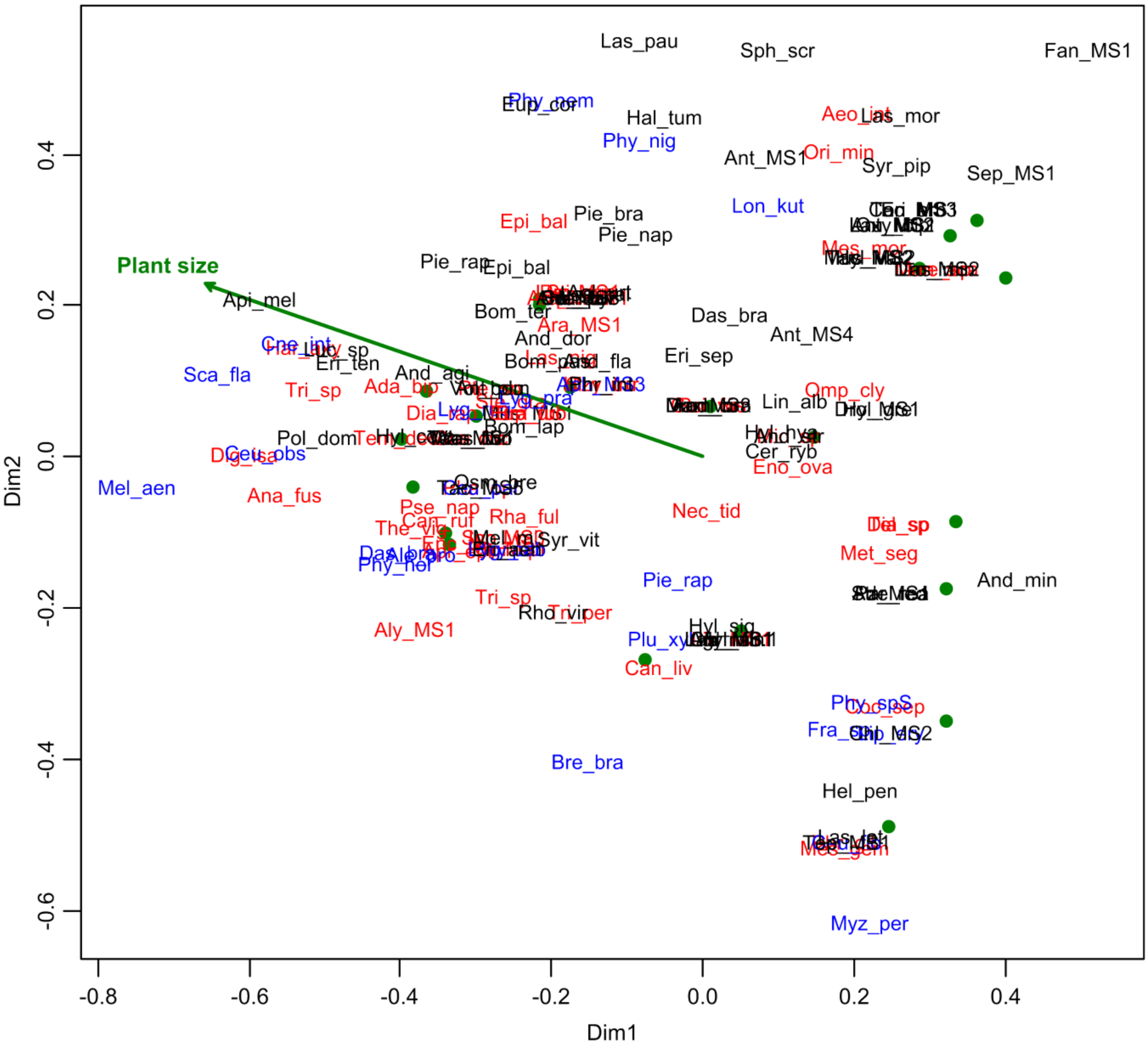

Figure S2. Community composition ordination (PCoA). The matrix of arthropod species (based on abundance data per plant species and Bray-Curtis dissimilarity; herbivore species in blue, their natural enemies in red and pollinator species in black; abbreviations refer to the first letters of the species names, see S3 Table) and plant species (green dots) was calculated independently of plant size (variance explained by axes = 40 % each). Association between ordination and plant size: $R^2 = 0.630$, $p < 0.001$.
