# Supplementary material for: Plant Size as Determinant of Species Richness of Herbivores, Natural Enemies and Pollinators across 21 Brassicaceae Species
Source: PLoS One. 2015 Aug 20;10(8):e0135928. doi: 10.1371/journal.pone.0135928 (PMC4546192; doi:10.1371/journal.pone.0135928)
Supplement: S1 File — (PDF) [file pone.0135928.s003.pdf]

Supporting Information S1 File: Abundance-based coverage estimators.

We computed the abundance-based coverage estimator (ACE) of herbivore and natural enemy species richness per plant species (see [1] for an overview), referring to the estimated species richness of every plant species under ideal sampling intensity using EstimateS [2]. These values of estimated species richness for herbivores and natural enemies (sqrt-transformed) were used as response variables within multiple regressions with plant size and, testing natural enemy species richness, estimated herbivore species richness (Pearson's correlation coefficient of the significant correlation between these explanatory variables: 0.73) and their two-way interaction as explanatory variables. Results show increasing estimated species richness of herbivores with increasing plant size (parameter weight = 1.00; estimate with SE of centred and standardised data = 3.763 ± 0.798, Fig. S1a), while estimated species richness of their natural enemies was positively related to estimated herbivore species richness (parameter weight = 0.57; estimate with SE = 0.454 ± 0.166, Fig. S1b) and not to plant size (parameter weight = 0.43) or the interaction of both (parameter weight = 0.00).

Percentage of observed from expected species richness (ACE) was not correlated with plant size (herbivores p = 0.439, cor = 0.178; natural enemies p = 0.514, cor = 0.151; both together p = 0.352, cor = 0.214).

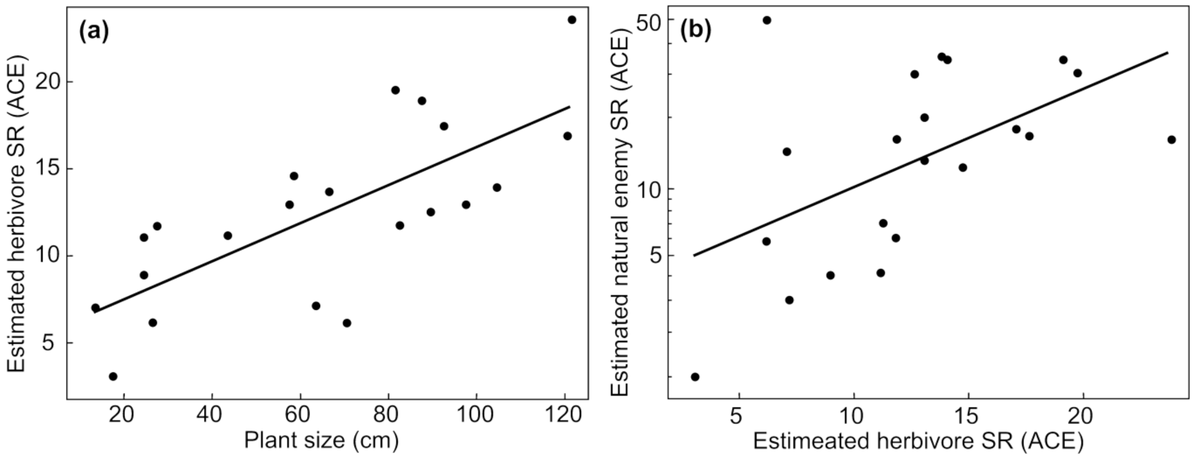

Figure S1. Effects of plant size and covariables on estimated species richness (ACE) of (a) herbivores and (b) their natural enemies. SR = species richness, H = herbivores, NE = natural enemies. Axes of variables were transformed corresponding to analyses (estimated species richness of natural enemies: log-transformation).

References

1. Magurran AE. Measuring biological diversity [Internet]. 2004. Available: http://www.tandfonline.com/doi/pdf/10.2989/16085910409503825
2. Colwell RK. EstimateS: Statistical estimation of species richness and shared species from samples. [Internet]. 2009. Available: http://viceroy.eeb.uconn.edu/estimates/
