# Supplementary material for: Plant Size as Determinant of Species Richness of Herbivores, Natural Enemies and Pollinators across 21 Brassicaceae Species
Source: PLoS One. 2015 Aug 20;10(8):e0135928. doi: 10.1371/journal.pone.0135928 (PMC4546192; doi:10.1371/journal.pone.0135928)
Supplement: S1 Table — (PDF) [file pone.0135928.s004.pdf]

Supporting Information S1 Table: Correlations between component size and other plant characteristics.

Table S1. Correlation coefficients (except for flower colour: Student's t) and levels of significance are given with *$p < 0.05$, **$p < 0.01$ and ***$p < 0.001$. Number and biomass of flowers refer to plot level, while number and biomass of leaves and fruits refer to five plant individuals per plot. Number and biomass of flowers, fruits and leaves, petal length and fruit size were log-transformed. Empty cells refer to combinations of variables which were not tested.

	Plant size (cm)	Flower number	Flower biomass (g)	Flower colour	Fruit number	Fruit biomass (g)	Leaf number	Leaf biomass (g)
Petal length (mm)	0.514*	-0.639**	0.442*	-2.796**				
Fruit size (mm^2)	0.464*				-0.835***	0.514*		
Leaf area (cm^2)	0.823***						ns	0.585**
