# Supplementary material for: Plant Size as Determinant of Species Richness of Herbivores, Natural Enemies and Pollinators across 21 Brassicaceae Species
Source: PLoS One. 2015 Aug 20;10(8):e0135928. doi: 10.1371/journal.pone.0135928 (PMC4546192; doi:10.1371/journal.pone.0135928)
Supplement: S2 Table — (PDF) [file pone.0135928.s005.pdf]

Supporting Information S2 Table: Means and standard errors per plant species of all tested parameters.

Table S2a. Means and standard errors per plant species of all tested parameters (based on 4 plots per plant species, data on flower biomass based on 2 plots per plant species as single exception); species richness = SR, *Diplotaxis muralis* = Dm, *Arabidopsis thaliana* = At, *Thlaspi arvense* = Ta, *Capsella bursa-pastoris* = Cb, *Lepidium virginicum* = Lv, *Lepidium campestre* = Lc, *Camelina sativa* = Cs. Number and biomass of flowers and pollinator species richness refer to plot level, while number and biomass of leaves and fruits and species richness of herbivores and their natural enemies refer to five plant individuals per plot.

	Dm	At	Ta	Cb	Lv	Lc	Cs
Herbivore SR	3.75 ± 0.25	0.50 ± 0.29	4.50 ± 1.19	3.00 ± 0.00	3.50 ± 0.29	4.75 ± 1.11	3.00 ± 0.41
Ectophagous	1.75 ± 0.48	0.25 ± 0.25	3.25 ± 0.75	2.75 ± 0.25	3.00 ± 0.41	4.50 ± 0.87	1.00 ± 0.41
Endophagous	2.25 ± 0.25	0.25 ± 0.25	1.25 ± 0.48	1.25 ± 0.25	1.00 ± 0.41	0.25 ± 0.25	2.00 ± 0.41
Leaves	1.25 ± 0.25	0.00 ± 0.00	1.25 ± 0.48	2.75 ± 0.25	1.50 ± 0.29	3.00 ± 0.41	1.00 ± 0.41
Fruits	2.25 ± 0.25	0.25 ± 0.25	1.00 ± 0.41	1.25 ± 0.25	0.50 ± 0.29	0.25 ± 0.25	1.25 ± 0.48
Natural enemy SR	3.00 ± 0.58	0.25 ± 0.25	0.75 ± 0.75	1.75 ± 0.48	2.25 ± 1.31	1.25 ± 0.48	1.25 ± 0.25
Ectophagous	0.00 ± 0.00	0.25 ± 0.25	0.50 ± 0.50	0.00 ± 0.00	1.50 ± 0.87	1.25 ± 0.48	0.25 ± 0.25
Endophagous	3.00 ± 0.58	0.00 ± 0.00	0.25 ± 0.25	1.75 ± 0.48	0.75 ± 0.48	0.00 ± 0.00	1.00 ± 0.00
Leaves	0.00 ± 0.00	0.00 ± 0.00	0.25 ± 0.25	0.00 ± 0.00	0.50 ± 0.29	1.00 ± 0.41	0.50 ± 0.29
Fruits	3.00 ± 0.58	0.00 ± 0.00	0.25 ± 0.25	1.75 ± 0.48	0.50 ± 0.29	0.00 ± 0.00	0.75 ± 0.25
Pollinator SR	3.00 ± 0.41	5.25 ± 1.03	3.75 ± 1.18	3.75 ± 1.49	5.00 ± 1.78	12.50 ± 2.18	4.25 ± 0.48
Plant size (cm)	12.65 ± 1.21	16.85 ± 0.44	24.00 ± 2.89	24.05 ± 0.66	26.05 ± 1.09	27.45 ± 1.69	42.75 ± 2.29
Number flowers	146.40 ± 16.56	2053.85 ± 138.54	557.05 ± 163.06	730.40 ± 139.04	10209.00 ± 1566.61	13213.25 ± 5718.94	532.00 ± 98.16
Biomass flowers (g)	12.75 ± 4.68	45.38 ± 9.46	3.79 ± 0.04	23.05 ± 4.07	3.58 ± 0.07	57.43 ± 18.81	12.88 ± 3.37
Flower colour	2.00 ± 0.00	1.00 ± 0.00	1.00 ± 0.00	1.00 ± 0.00	1.00 ± 0.00	1.00 ± 0.00	2.00 ± 0.00
Petal length (mm)	7.35 ± 0.22	4.10 ± 0.06	4.15 ± 0.10	3.20 ± 0.12	1.00 ± 0.00	2.53 ± 0.34	6.40 ± 0.18
Number leaves	231.00 ± 9.50	29.25 ± 13.52	180.75 ± 20.42	206.25 ± 24.12	784.50 ± 151.11	259.75 ± 45.37	900.00 ± 89.61
Biomass leaves (g)	2.79 ± 1.21	0.04 ± 0.02	0.87 ± 0.24	0.81 ± 0.20	3.00 ± 0.90	3.07 ± 0.98	0.93 ± 0.26
Leaf area (cm^2)	11.50 ± 0.67	0.40 ± 0.08	10.30 ± 1.21	4.60 ± 0.67	4.85 ± 0.62	2.25 ± 0.10	15.50 ± 2.08
Number fruits	834.25 ± 101.37	3387.50 ± 1378.61	1277.25 ± 90.83	5262.50 ± 569.13	10105.00 ± 3621.89	5067.50 ± 681.51	1400.00 ± 207.81
Biomass fruits (g)	15.20 ± 4.27	10.17 ± 2.13	10.66 ± 2.44	11.34 ± 2.56	9.55 ± 1.21	29.84 ± 7.12	28.22 ± 4.84
Fruit size (mm^2)	56.95 ± 3.77	10.70 ± 1.30	151.10 ± 6.11	31.80 ± 1.53	5.66 ± 0.49	19.54 ± 0.25	39.39 ± 1.33

Table S2b. Means and standard errors per plant species of all tested parameters (based on 4 plots per plant species, data on flower biomass based on 2 plots per plant species as single exception); species richness = SR, *Sinapis arvensis* = Sar, *Sinapis alba* = Sal, *Lepidium sativum* = Ls, *Raphanus raphanistrum* = Rra, *Sisymbrium officinale* = So, *Descurainia sophia* = Ds, *Eruca sativa* = Es. Number and biomass of flowers and pollinator species richness refer to plot level, while number and biomass of leaves and fruits and species richness of herbivores and their natural enemies refer to five plant individuals per plot.

	Sar	Sal	Ls	Rra	So	Ds	Es
Herbivore SR	7.00 ± 1.29	4.75 ± 0.63	3.50 ± 0.65	6.00 ± 0.41	3.50 ± 0.29	6.00 ± 1.08	7.50 ± 0.65
Ectophagous	5.25 ± 0.95	3.75 ± 0.63	3.50 ± 0.65	3.75 ± 0.48	3.50 ± 0.29	5.00 ± 0.82	4.25 ± 0.48
Endophagous	2.50 ± 0.50	1.50 ± 0.29	0.25 ± 0.25	3.00 ± 0.71	1.00 ± 0.41	1.50 ± 0.65	4.00 ± 0.58
Leaves	3.00 ± 1.15	2.50 ± 0.65	1.25 ± 0.48	3.00 ± 0.41	1.50 ± 0.29	4.00 ± 0.58	4.75 ± 1.03
Fruits	1.75 ± 0.25	0.75 ± 0.25	0.25 ± 0.25	2.00 ± 0.41	0.75 ± 0.25	0.75 ± 0.48	2.00 ± 0.00
Natural enemy SR	3.50 ± 1.26	2.00 ± 0.71	0.50 ± 0.29	4.75 ± 0.95	1.75 ± 0.85	3.00 ± 0.91	4.00 ± 0.41
Ectophagous	1.25 ± 0.63	1.25 ± 0.63	0.50 ± 0.29	1.75 ± 0.48	0.75 ± 0.48	2.50 ± 0.65	1.50 ± 0.29
Endophagous	2.25 ± 0.63	0.75 ± 0.25	0.00 ± 0.00	3.00 ± 0.91	1.00 ± 0.41	0.50 ± 0.29	2.50 ± 0.29
Leaves	0.75 ± 0.48	0.00 ± 0.00	0.25 ± 0.25	1.25 ± 0.25	0.00 ± 0.00	2.75 ± 0.85	1.50 ± 0.29
Fruits	2.25 ± 0.63	0.75 ± 0.25	0.00 ± 0.00	2.25 ± 0.63	1.00 ± 0.41	0.25 ± 0.25	2.25 ± 0.25
Pollinator SR	8.75 ± 1.03	10.25 ± 1.11	12.25 ± 0.75	9.50 ± 0.65	10.25 ± 1.11	12.25 ± 1.31	12.00 ± 0.41
Plant size (cm)	56.50 ± 1.26	58.00 ± 4.30	63.25 ± 1.38	65.70 ± 6.53	69.80 ± 3.10	80.55 ± 4.20	82.15 ± 2.07
Number flowers	580.20 ± 60.05	776.45 ± 175.44	16478.50 ± 2670.06	275.15 ± 132.66	5698.00 ± 1452.55	4883.30 ± 469.29	620.05 ± 77.39
Biomass flowers (g)	37.07 ± 11.10	21.47 ± 4.10	77.51 ± 1.66	35.79 ± 13.40	10.11 ± 3.49	5.52 ± 1.31	36.30 ± 6.17
Flower colour	2.00 ± 0.00	2.00 ± 0.00	1.00 ± 0.00	1.00 ± 0.00	2.00 ± 0.00	2.00 ± 0.00	1.00 ± 0.00
Petal length (mm)	11.65 ± 0.26	11.70 ± 0.17	4.05 ± 0.05	16.65 ± 0.94	4.53 ± 0.18	2.00 ± 0.00	23.80 ± 0.95
Number leaves	119.00 ± 20.52	164.75 ± 6.07	387.75 ± 35.97	166.25 ± 29.21	177.25 ± 33.66	126.00 ± 20.69	130.00 ± 19.20
Biomass leaves (g)	1.47 ± 0.49	3.40 ± 0.51	1.43 ± 0.26	5.95 ± 1.59	1.28 ± 0.34	2.30 ± 0.86	6.67 ± 1.57
Leaf area (cm^2)	65.50 ± 9.76	33.35 ± 5.25	25.30 ± 4.75	53.70 ± 10.83	8.90 ± 1.11	5.45 ± 0.94	30.05 ± 3.13
Number fruits	1045.00 ± 293.02	770.00 ± 120.68	5805.00 ± 687.53	744.00 ± 266.26	1430.50 ± 249.18	1572.25 ± 142.91	470.75 ± 57.77
Biomass fruits (g)	52.88 ± 15.49	56.02 ± 11.09	51.32 ± 5.16	14.45 ± 8.00	15.74 ± 3.31	5.14 ± 1.44	33.79 ± 8.70
Fruit size (mm^2)	90.75 ± 7.67	145.10 ± 4.46	27.30 ± 1.03	137.73 ± 20.26	17.83 ± 1.24	24.50 ± 0.51	121.75 ± 8.66

Table S2c. Means and standard errors per plant species of all tested parameters (based on 4 plots per plant species, data on flower biomass based on 2 plots per plant species as single exception); species richness = SR, *Sisymbrium loeselii* = Sl, *Brassica nigra* = Bni, *Rapistrum rugosum* = Rru, *Brassica napus* = Bna, *Raphanus sativus sativus* = Rss, *Brassica juncea* = Bj, *Raphanus sativus oleiformis* = Rso. Number and biomass of flowers and pollinator species richness refer to plot level, while number and biomass of leaves and fruits and species richness of herbivores and their natural enemies refer to five plant individuals per plot.

	Sl	Bni	Rru	Bna	Rss	Bj	Rso
Herbivore SR	6.50 ± 0.50	8.25 ± 1.11	9.00 ± 0.82	6.75 ± 0.48	5.25 ± 0.63	8.75 ± 1.49	7.00 ± 0.71
Ectophagous	5.25 ± 0.25	5.00 ± 0.58	6.25 ± 0.85	4.25 ± 0.63	3.00 ± 0.00	6.00 ± 1.08	4.50 ± 1.19
Endophagous	2.50 ± 0.50	4.25 ± 0.85	4.00 ± 0.41	3.75 ± 0.48	3.25 ± 0.63	4.00 ± 0.58	3.50 ± 0.87
Leaves	4.50 ± 0.29	5.00 ± 1.08	4.25 ± 0.95	4.00 ± 0.41	3.00 ± 0.71	6.00 ± 1.29	3.25 ± 0.63
Fruits	1.50 ± 0.29	2.00 ± 0.00	2.00 ± 0.00	2.25 ± 0.25	1.50 ± 0.50	2.00 ± 0.00	2.25 ± 0.25
Natural enemy SR	4.00 ± 0.58	5.75 ± 1.49	5.00 ± 0.41	5.50 ± 0.65	4.00 ± 0.91	5.50 ± 1.19	6.00 ± 0.71
Ectophagous	2.00 ± 0.41	1.50 ± 0.87	1.25 ± 0.25	1.50 ± 0.50	1.25 ± 0.63	3.00 ± 0.91	0.50 ± 0.29
Endophagous	2.00 ± 0.41	4.25 ± 1.31	3.75 ± 0.48	4.00 ± 0.41	2.75 ± 0.48	2.50 ± 0.50	5.50 ± 0.65
Leaves	2.25 ± 0.48	1.75 ± 0.85	2.75 ± 0.25	1.50 ± 0.65	0.75 ± 0.25	2.50 ± 0.65	1.00 ± 0.41
Fruits	1.25 ± 0.25	3.50 ± 0.65	2.00 ± 0.00	3.50 ± 0.29	2.75 ± 0.48	2.00 ± 0.00	5.00 ± 0.58
Pollinator SR	10.50 ± 1.55	5.25 ± 0.48	12.00 ± 1.83	9.25 ± 1.97	11.75 ± 1.11	8.50 ± 1.19	8.25 ± 0.75
Plant size (cm)	86.50 ± 4.73	88.50 ± 2.90	91.65 ± 1.69	96.75 ± 5.51	104.15 ± 2.99	119.80 ± 2.85	120.50 ± 3.40
Number flowers	3311.75 ± 1304.32	427.05 ± 47.24	4196.10 ± 962.85	200.50 ± 56.62	827.35 ± 173.53	342.30 ± 225.48	229.45 ± 31.55
Biomass flowers (g)	15.32 ± 2.51	23.41 ± 5.08	34.92 ± 3.85	22.98 ± 3.79	17.85 ± 4.44	32.27 ± 1.22	53.55 ± 9.76
Flower colour	2.00 ± 0.00	2.00 ± 0.00	2.00 ± 0.00	2.00 ± 0.00	1.00 ± 0.00	2.00 ± 0.00	1.00 ± 0.00
Petal length (mm)	5.30 ± 0.24	6.95 ± 0.05	7.20 ± 0.22	10.45 ± 0.39	16.80 ± 1.49	6.95 ± 0.46	15.10 ± 0.39
Number leaves	222.50 ± 34.26	225.00 ± 28.59	396.00 ± 53.14	160.00 ± 21.12	156.00 ± 16.35	117.75 ± 6.49	164.00 ± 5.76
Biomass leaves (g)	13.70 ± 2.21	5.16 ± 2.13	14.90 ± 2.66	13.14 ± 2.75	3.62 ± 0.31	9.62 ± 1.77	3.04 ± 1.41
Leaf area (cm²)	36.45 ± 4.07	75.30 ± 8.58	62.40 ± 8.25	76.85 ± 11.26	68.35 ± 7.68	91.70 ± 7.95	91.65 ± 14.16
Number fruits	3379.50 ± 702.53	825.75 ± 130.88	5158.75 ± 808.33	498.25 ± 83.33	336.25 ± 29.88	776.25 ± 412.72	847.50 ± 241.77
Biomass fruits (g)	12.56 ± 3.76	35.32 ± 2.27	38.42 ± 4.23	27.95 ± 1.15	41.78 ± 4.24	7.38 ± 1.69	96.59 ± 25.14
Fruit size (mm²)	22.00 ± 1.19	111.05 ± 6.88	19.45 ± 1.09	198.15 ± 12.86	276.00 ± 8.05	53.05 ± 3.40	409.90 ± 47.07
