# Supplementary material for: Plant Size as Determinant of Species Richness of Herbivores, Natural Enemies and Pollinators across 21 Brassicaceae Species
Source: PLoS One. 2015 Aug 20;10(8):e0135928. doi: 10.1371/journal.pone.0135928 (PMC4546192; doi:10.1371/journal.pone.0135928)
Supplement: S3 Table — (PDF) [file pone.0135928.s006.pdf]

Supporting Information S3 Table: Species list.

Table S3. Species list of observed individuals (herbivore species in blue, their natural enemies in red and pollinator species in black). MS = morphospecies.

	Species	Order
Herbivores	*Ceutorhynchus floralis* (Paykull 1792)	Coleoptera
	Ceutorhynchus obstrictus (Marsham 1802)	Coleoptera
	Ceutorhynchus pallidactylus (Marsham 1802)	Coleoptera
	Longitarsus kutscherae (Rye 1872)	Coleoptera
	Meligethes aeneus (Fabricius 1775)	Coleoptera
	Phyllotreta atra (Fabricius 1775)	Coleoptera
	Phyllotreta nemorum (Linnaeus 1758)	Coleoptera
	Phyllotreta nigripes (Fabricius 1775)	Coleoptera
	Dasineura brassicae Winnertz 1853	Diptera
	Phytomyza horticola Goureau 1851	Diptera
	Phytomyza sp. MS1	Diptera
	Scaptomyza flava Fallen 1823	Diptera
	Aleyrodes proletella Linnaeus 1758	Hemiptera
	Aphidoidea MS3	Hemiptera
	Brevicoryne brassicae (Linnaeus 1758)	Hemiptera
	Lipaphis cf. *erysimi* (Kaltenbach 1843)	Hemiptera
	Lygocoris pabulinus (Linnaeus 1761)	Hemiptera
	Lygus pratensis (Linnaeus 1758)	Hemiptera
	Lygus rugulipennis Poppius 1911	Hemiptera
	Myzus persicae (Sulzer 1776)	Hemiptera
	Cnephasia interjectana (Haworth 1811)	Lepidoptera
	Pieris rapae Linnaeus 1758	Lepidoptera
	Plutella xylostella (Linnaeus 1758)	Lepidoptera
	Frankliniella sp.	Thysanoptera
natural enemies	Araneae MS1	Araneae
	Araneidae MS1	Araneae
	Araneus diadematus Clerck 1757	Araneae
	cf. *Anelosimus* sp.	Araneae
	cf. *Mangora acalypha* (Walckenaer 1802)	Araneae
	Enoplognatha ovata (Clerck 1757)	Araneae
	Linyphiidae MS1	Araneae
	Lycosidae MS1	Araneae
	Metellina segmentata (Clerck 1787)	Araneae
	Ozyptila cf. *trux* (Blackwall 1846)	Araneae
	Adalia bipunctata (Linnaeus 1758)	Coleoptera
	Cantharis bicolor Linnaeus 1763	Coleoptera
	Cantharis fusca Linnaeus 1758	Coleoptera
	Cantharis livida Linnaeus 1758	Coleoptera
	Cantharis rufa Linnaeus 1758	Coleoptera
	Coccinella septempunctata Linnaeus 1758	Coleoptera
	Harmonia axyridis Pallas 1773	Coleoptera
	Propylaea quatuordecimpunctata (Linnaeus 1758)	Coleoptera
	Psilothrix viridicoeruleus (Geoffroy in Fourcroy 1785)	Coleoptera
	Rhagonycha fulva Scopoli 1763	Coleoptera
	Thea vigintiduopunctata Moscardini 1954	Coleoptera
	Episyrphus balteatus (de Geer 1776)	Diptera
	Syrphidae MS3	Diptera
	Himacerus mirmicoides (O. Costa 1834)	Hemiptera
	Orius minutus (Linneaus 1758)	Hemiptera
	Alysiinae MS1	Hymenoptera
	Anaphes fuscipennis Haliday 1833	Hymenoptera
	Aprostocetus epicharmus Walker 1839	Hymenoptera
	Bracon fulvipes Nees 1834	Hymenoptera
	Diadegma sp.	Hymenoptera
	Diaeretiella rapae (McIntosh 1855)	Hymenoptera
	Diglyphus isaea (Walker 1838)	Hymenoptera

	Species	Order
natural enemies (continuation)	*Encarsia tricolor* Forster 1878	Hymenoptera
	Eupelmus urozonus Dalman 1821	Hymenoptera
	Ichneumoninae MS1	Hymenoptera
	Inostemma walkeri Kieffer 1914	Hymenoptera
	Lasius niger (Linnaeus 1758)	Hymenoptera
	Mesopolobus gemellus Baur Muller 2007	Hymenoptera
	Mesopolobus morys Walker 1848	Hymenoptera
	Microplitis sp.	Hymenoptera
	Necremnus tidius (Walker 1839)	Hymenoptera
	Omphale clypealis (Thomson 1878)	Hymenoptera
	Opius sp.	Hymenoptera
	Phradis gibbus Holmgren 1860	Hymenoptera
	Pseudotorymus napi Amerling Kirchner 1860	Hymenoptera
	Pteromalus sp.	Hymenoptera
	Stenomalina gracilis Walker 1834	Hymenoptera
	Stibeutes curvispina Thomson 1884	Hymenoptera
	Telenomus sp.	Hymenoptera
	Temelucha decorata Gravenhorst 1829	Hymenoptera
	Trichogramma sp.	Hymenoptera
	Trichomalus perfectus Walker 1835	Hymenoptera
	Trichopria sp.	Hymenoptera
	Opilio canestrinii (Thorell 1876)	Opiliones
	Phalangium opilio Linnaeus 1761	Opiliones
	Aeolothrips intermedius Bagnall 1934	Thysanoptera
pollinators	Agromyzidae MS1	Diptera
	Anthomyiidae MS1	Diptera
	Anthomyiidae MS2	Diptera
	Anthomyiidae MS3	Diptera
	Anthomyiidae MS4	Diptera
	Chloropidae MS1	Diptera
	Chloropidae MS2	Diptera
	Conopidae MS1	Diptera
	Dasineura brassicae Winnertz 1853	Diptera
	Drosophilidae MS1	Diptera
	Drosophilidae MS2	Diptera
	Drosophilidae MS3	Diptera
	Episyrphus balteatus (de Geer 1776)	Diptera
	Eristalinus aeneus (Scopoli 1763)	Diptera
	Eristalinus sepulchralis (Linnaeus 1758)	Diptera
	Eristalis arbustorum (Linnaeus 1758)	Diptera
	Eristalis tenax (Linnaeus 1758)	Diptera
	Eupeodes corollae (Fabricius 1794)	Diptera
	Fanniidae MS1	Diptera
	Helophilus pendulus (Linnaeus 1758)	Diptera
	Helophilus trivittatus (Fabricius 1805)	Diptera
	Lauxaniidae MS1	Diptera
	Lucilia sp.	Diptera
	Melanostoma mellinum (Linnaeus 1758)	Diptera
	Muscidae MS1	Diptera
	Muscidae MS2	Diptera
	Scaeva pyrastri (Linnaeus 1758)	Diptera
	Sepsidae MS1	Diptera
	Sphaerophoria scripta Linnaeus 1758	Diptera
	Stratiomyidae MS1	Diptera
	Syritta pipiens Linnaeus 1758	Diptera
	Syrphus vitripennis Meigen 1822	Diptera
	Tachinidae MS1	Diptera
	Tachinidae MS2	Diptera
	Tachinidae MS3	Diptera
	Tachinidae MS5	Diptera
	Tephritidae MS1	Diptera

	Species	Order
pollinators (continuation)	*Volucella bombylans* (Linnaeus 1758)	Diptera
	Andrena agilissima (Scopoli 1770)	Hymenoptera
	Andrena cf. *minutuloides* Perkins 1914	Hymenoptera
	Andrena cineraria (Linnaeus 1758)	Hymenoptera
	Andrena dorsata (Kirby 1802)	Hymenoptera
	Andrena flavipes Panzer 1799	Hymenoptera
	Andrena strohmella Illiger 1806	Hymenoptera
	Anthophora plumipes (Pallas 1772)	Hymenoptera
	Apis mellifera Linnaeus 1758	Hymenoptera
	Bombus lapidarius Linnaeus 1758	Hymenoptera
	Bombus pascuorum Scopoli 1763	Hymenoptera
	Bombus terrestris (Linnaeus 1758)	Hymenoptera
	Cerceris rybyensis (Linnaeus 1771)	Hymenoptera
	Colletes daviesanus Smith 1846	Hymenoptera
	Halictus tumulorum (Linnaeus 1758)	Hymenoptera
	Hylaeus communis Cookerell 1937	Hymenoptera
	Hylaeus gredleri Foerster 1871	Hymenoptera
	Hylaeus hyalinatus Smith 1843	Hymenoptera
	Hylaeus signatus (Panzer 1798)	Hymenoptera
	Hylaeus sinuatus (Schenck 1853)	Hymenoptera
	Hylaeus variegatus (Fabricius 1798)	Hymenoptera
	Lasioglossum calceatum (Scopoli 1763)	Hymenoptera
	Lasioglossum laticeps Schenk 1870	Hymenoptera
	Lasioglossum minutissimum Kirby 1802	Hymenoptera
	Lasioglossum minutulum (Schenck 1853)	Hymenoptera
	Lasioglossum morio (Fabricius 1793)	Hymenoptera
	Lasioglossum pauxillum (Schenck 1853)	Hymenoptera
	Lindenius albilabris (Fabricius 1793)	Hymenoptera
	Osmia brevicornis (Fabricius 1798)	Hymenoptera
	Oxybelus bipunctatus Olivier 1812	Hymenoptera
	Phradis interstitialis (Thomson 1889)	Hymenoptera
	Polistes dominulus (Christ 1791)	Hymenoptera
	Rhogogaster viridis (Linnaeus 1758)	Hymenoptera
	Terebrantia MS1	Hymenoptera
	Vespidae MS1	Hymenoptera
	Vespidae MS2	Hymenoptera
	Adela reaumurella Linnaeus 1758	Lepidoptera
	Aglais urticae Linnaeus 1758	Lepidoptera
	Mamestra brassicae (Linnaeus 1758)	Lepidoptera
	Pieris brassicae Linnaeus 1758	Lepidoptera
	Pieris napi Linnaeus 1758	Lepidoptera
	Pieris rapae Linnaeus 1758	Lepidoptera
