# Supplementary material for: Plant Size as Determinant of Species Richness of Herbivores, Natural Enemies and Pollinators across 21 Brassicaceae Species
Source: PLoS One. 2015 Aug 20;10(8):e0135928. doi: 10.1371/journal.pone.0135928 (PMC4546192; doi:10.1371/journal.pone.0135928)
Supplement: S4 Table — (PDF) [file pone.0135928.s007.pdf]

Supporting Information S4 Table: Raw data.

Table S4. Raw data of all tested parameters, species richness = SR. Number and biomass of flowers and pollinator species richness refer to plot level, while number and biomass of leaves and fruits and species richness and abundance of herbivores and their natural enemies refer to five plant individuals per plot.

Plot number	Plant species	Overall SR	SR Herbivores	SR ectophagous herbivores	SR endophagous herbivores	SR leaf associated herbivores	SR fruit associated herbivores	Abundance herbivores	Abundance ectophagous herbivores	Abundance endophagous herbivores	Abundance leaf associated herbivores	Abundance fruit associated herbivores	SR natural enemies of herbivores	SR natural enemies of ectophagous herbivores	SR natural enemies of endophagous herbivores	SR natural enemies of leaf associated herbivores	SR natural enemies of fruit associated herbivores	SR pollinators	Plant size (cm)	Number flowers	Biomass flowers (g)	Flower colour	Petal length (mm)	Number leaves	Biomass leaves (g)	Leaf area (cm²)	Number fruits	Biomass fruits (g)	Fruit size (mm²)
1	*Arabidopsis thaliana*	7	0	0	0	0	0	0	0	0	0	0	0	0	0	0	0	7	16	2237	NA	white	4	69	0.00	1	6800	14.46	12
2	*Capsella bursa-pastoris*	9	3	3	1	3	1	130	58	72	10	72	3	0	3	0	3	3	26	1094	NA	white	3	201	0.96	4	6200	7.70	30
3	*Raphanus raphanistrum*	20	6	3	4	3	3	220	174	46	15	33	6	1	5	1	4	8	47	659	49	white	17	91	7.93	26	183	1.62	182
4	*Raphanus sativus sativus*	22	5	3	3	3	1	105	96	9	2	8	6	3	3	1	3	11	99	1188	13	white	15	141	2.90	45	292	29.66	289
5	*Raphanus raphanistrum*	17	5	4	1	2	1	106	105	1	4	1	2	1	1	1	1	10	72	134	NA	white	14	204	3.22	55	445	5.65	157
6	*Descurainia sophia*	23	7	5	3	5	1	56	54	2	23	1	5	4	1	5	0	12	93	6240	NA	yellow	2	93	4.86	3	1644	9.15	25
7	*Lepidium campestre*	29	8	7	1	4	1	17	16	1	11	1	2	2	0	2	0	19	26	6232	76	white	3	168	5.91	2	6320	18.84	19
8	*Diplotaxis muralis*	11	4	1	3	1	3	57	2	55	2	55	4	0	4	0	4	3	14	132	NA	yellow	7	217	6.32	12	935	18.67	58
9	*Eruca sativa*	23	6	3	3	2	2	207	202	5	2	4	5	2	3	2	3	12	78	538	30	white	21	76	3.70	25	345	17.78	109
10	*Sinapis alba*	15	3	2	1	1	0	64	64	0	0	0	1	1	0	0	0	11	56	1008	26	yellow	11	153	4.32	28	480	30.61	135
11	*Lepidium sativum*	16	2	2	0	0	0	4	4	0	0	0	1	1	0	1	0	13	66	23380	79	white	4	329	1.31	14	6950	48.89	30
12	*Sinapis arvensis*	11	4	4	1	1	1	43	42	1	7	1	1	0	1	0	1	6	57	638	NA	yellow	11	68	2.37	37	635	28.16	68
14	*Descurainia sophia*	20	3	3	0	3	0	17	17	0	5	0	1	1	0	1	0	16	75	4563	NA	yellow	2	106	1.54	5	1160	2.30	24
15	*Raphanus sativus sativus*	25	7	3	5	4	3	115	101	14	11	7	5	1	4	1	4	13	104	353	NA	white	14	140	3.74	78	290	47.29	255
16	*Lepidium campestre*	16	4	4	0	3	0	15	15	0	6	0	2	2	0	1	0	10	32	10850	NA	white	2	256	1.55	2	3520	27.59	19

17	*Brassica juncea*	25	10	7	5	7	2	574	541	33	35	14	4	2	2	2	2	11	119	106	33	yellow	6	106	11.07	81	615	5.94	62
19	*Sisymbrium officinale*	16	4	4	2	2	1	19	17	2	7	2	4	2	2	0	2	8	66	1722	7	yellow	5	140	1.96	6	1987	25.44	17
20	*Sinapis arvensis*	20	8	5	3	5	2	40	36	4	26	4	3	1	2	0	2	9	53	594	NA	yellow	12	105	1.96	68	745	45.79	97
21	*Lepidium campestre*	15	3	3	0	2	0	10	10	0	7	0	1	1	0	1	0	11	26	5751	NA	white	3	384	2.13	2	6100	50.51	20
22	*Brassica nigra*	20	9	6	4	5	2	1092	1075	17	58	13	7	4	3	2	3	4	91	516	NA	yellow	7	223	3.88	63	1113	37.15	105
23	*Sisymbrium loeselii*	18	8	5	4	5	2	80	52	28	26	5	5	2	3	3	2	6	79	638	NA	yellow	6	162	8.23	26	1302	6.09	19
24	*Sinapis alba*	15	5	5	1	3	1	39	38	1	3	1	1	0	1	0	1	9	47	504	17	yellow	12	156	2.51	25	725	45.24	140
25	*Brassica juncea*	18	5	3	3	3	2	363	351	12	9	8	7	5	2	3	2	7	115	225	NA	yellow	8	112	9.40	104	1984	12.21	55
27	*Rapistrum rugosum*	22	7	4	4	3	2	856	830	26	8	21	6	1	5	3	2	10	87	1354	31	yellow	7	281	13.49	41	6890	37.51	17
28	*Brassica napus*	23	6	4	3	3	2	317	194	123	24	105	4	0	4	0	4	14	113	138	19	yellow	10	118	21.01	51	691	27.07	215
29	*Sisymbrium loeselii*	21	6	6	2	5	1	131	127	4	19	3	3	1	2	2	1	12	78	1521	NA	yellow	6	205	17.83	39	3970	23.11	25
30	*Sinapis alba*	16	6	4	2	4	1	66	64	2	4	2	2	1	1	0	1	8	62	450	NA	yellow	12	172	2.53	32	810	79.64	151
31	*Camelina sativa*	9	4	1	3	1	2	5	1	4	1	3	1	0	1	0	1	4	36	512	10	yellow	6	755	0.19	9	2010	39.98	37
32	*Brassica nigra*	15	5	4	2	2	2	187	171	16	89	16	5	1	4	1	4	5	90	301	18	yellow	7	161	0.55	61	980	29.00	108
33	*Raphanus sativus sativus*	22	5	3	3	4	1	53	43	10	4	9	3	1	2	1	2	14	112	894	NA	white	21	138	3.43	75	346	42.26	288
34	*Diplotaxis muralis*	7	3	1	2	1	2	12	1	11	1	11	2	0	2	0	2	2	10	138	NA	yellow	7	252	1.60	10	644	2.58	55
35	*Brassica juncea*	21	8	6	3	5	2	280	238	42	15	34	3	1	2	1	2	10	117	31	NA	yellow	7	117	4.84	107	330	4.44	46
38	*Camelina sativa*	10	3	1	2	0	2	3	1	2	0	2	2	1	1	1	1	5	46	294	16	yellow	7	735	1.39	17	1200	18.00	43
39	*Arabidopsis thaliana*	8	1	1	0	0	0	1	1	0	0	0	0	0	0	0	0	7	17	2250	55	white	4	23	0.01	0	1050	6.76	7
41	*Eruca sativa*	25	9	5	5	7	2	95	85	10	22	2	4	2	2	1	2	12	85	696	42	white	24	152	11.08	26	488	35.16	117
42	*Brassica napus*	19	6	3	4	4	2	266	146	120	111	18	7	2	5	3	4	6	94	346	27	yellow	10	133	12.79	68	375	26.86	209
43	*Brassica nigra*	18	10	6	5	6	2	149	141	8	115	6	2	0	2	0	2	6	80	479	NA	yellow	7	300	5.44	99	635	39.65	101
45	*Raphanus sativus sativus*	15	4	3	2	1	1	94	90	4	1	3	2	0	2	0	2	9	101	874	22	white	17	205	4.40	75	417	47.92	273
46	*Lepidium sativum*	18	5	5	0	2	0	584	584	0	20	0	0	0	0	0	0	13	60	17877	76	white	4	380	0.76	24	3810	63.58	26
47	*Lepidium virginicum*	15	4	3	2	2	1	12	9	3	5	2	6	4	2	1	5	5	25	9600	NA	white	1	554	4.54	6	5090	9.02	4
48	*Arabidopsis thaliana*	4	0	0	0	0	0	0	0	0	0	0	0	0	0	0	0	4	18	2072	NA	white	4	10	0.10	0	1250	6.24	11
50	*Lepidium virginicum*	15	4	4	1	1	0	19	18	1	2	0	1	1	0	0	0	10	26	14758	NA	white	1	1173	4.40	6	20600	13.12	7
51	*Eruca sativa*	22	7	5	3	5	2	377	370	7	16	5	4	1	3	2	2	11	86	451	NA	white	25	130	5.74	31	620	57.58	147
52	*Sisymbrium officinale*	18	4	4	1	2	1	27	26	1	4	1	1	0	1	0	1	13	79	7756	NA	yellow	4	141	1.75	9	1500	13.82	18
53	*Sinapis arvensis*	18	6	4	3	1	2	19	14	5	4	4	3	1	2	1	2	9	57	680	26	yellow	12	143	1.45	81	890	39.55	95
54	*Lepidium sativum*	14	4	4	1	1	1	20	19	1	1	1	0	0	0	0	0	10	62	11457	NA	white	4	351	1.90	25	6330	38.81	28
55	*Raphanus raphanistrum*	23	6	3	4	4	2	408	379	29	29	4	6	2	4	2	2	11	77	68	22	white	18	220	3.26	54	978	13.19	122
57	*Raphanus raphanistrum*	21	7	5	3	3	2	439	427	12	21	6	5	3	2	1	2	9	67	239	NA	white	18	150	9.39	79	1370	37.34	90
58	*Sinapis arvensis*	28	10	8	3	5	2	143	121	22	84	21	7	3	4	2	4	11	59	408	48	yellow	11	160	0.10	75	1910	98.03	103
59	*Rapistrum rugosum*	28	9	6	4	4	2	623	608	15	16	12	5	1	4	3	2	14	95	5621	NA	yellow	8	502	20.93	78	6175	48.81	22

60	*Raphanus sativus oleiformes*	23	7	2	6	3	3	127	74	53	4	48	7	0	7	1	6	9	118	216	63	white	16	147	7.11	50	1532	168.71	398
61	*Capsella bursa-pastoris*	5	3	2	2	2	2	284	252	32	4	32	1	0	1	0	1	1	23	800	NA	white	3	240	0.41	5	3650	7.90	36
62	*Brassica napus*	18	7	6	3	4	2	523	455	68	42	61	5	2	3	2	3	6	90	88	NA	yellow	10	179	9.93	84	583	26.50	209
64	*Raphanus sativus oleiformes*	15	5	3	3	3	2	174	148	26	5	25	4	0	4	0	4	6	114	146	NA	white	15	167	2.46	100	640	55.18	324
66	*Eruca sativa*	23	8	4	5	5	2	175	167	8	7	4	3	1	2	1	2	13	79	796	NA	white	25	162	6.16	39	430	24.66	114
67	*Brassica napus*	25	8	4	5	5	3	365	155	210	197	27	6	2	4	1	3	11	90	230	NA	yellow	12	210	8.82	104	344	31.38	160
68	*Diplotaxis muralis*	10	4	3	2	2	2	40	14	26	3	26	2	0	2	0	2	4	15	195	17	yellow	8	213	0.89	12	1070	21.46	48
69	*Thlaspi arvense*	6	2	2	0	1	0	12	12	0	1	0	0	0	0	0	0	4	29	999	4	white	4	154	0.85	12	1019	7.25	133
71	*Sisymbrium loeselii*	24	6	5	2	4	1	183	172	11	31	10	5	3	2	3	1	13	97	5760	18	yellow	5	321	16.69	46	3847	12.55	21
72	*Thlaspi arvense*	9	7	5	2	2	1	66	62	4	2	2	0	0	0	0	0	2	19	361	4	white	4	145	1.48	7	1300	8.27	156
73	*Descurainia sophia*	22	8	7	2	5	2	56	53	3	22	3	4	3	1	3	1	10	77	4080	7	yellow	2	119	1.27	6	1820	4.28	24
74	*Camelina sativa*	7	3	2	1	2	0	4	3	1	2	0	1	0	1	1	0	3	44	774	NA	yellow	6	1050	1.20	17	1090	23.13	41
75	*Rapistrum rugosum*	29	9	7	3	3	2	544	530	14	10	9	4	1	3	2	2	16	92	4833	NA	yellow	7	332	8.34	58	3920	28.15	18
76	*Lepidium sativum*	17	3	3	0	2	0	5	5	0	2	0	1	1	0	0	0	13	65	13200	NA	white	4	491	1.76	37	6130	54.01	26
78	*Capsella bursa-pastoris*	8	3	3	1	3	1	339	245	94	5	94	2	0	2	0	2	3	23	499	19	white	3	140	1.29	3	5300	11.12	30
80	*Capsella bursa-pastoris*	12	3	3	1	3	1	83	19	64	5	64	1	0	1	0	1	8	25	528	27	white	3	244	0.59	6	5900	18.66	31
81	*Sisymbrium loeselii*	19	6	5	2	4	2	173	170	3	31	3	3	2	1	1	1	11	92	5328	13	yellow	5	202	12.06	35	4399	8.51	23
82	*Lepidium virginicum*	7	3	2	1	2	1	8	7	1	4	1	2	1	1	1	1	2	25	7680	4	white	1	536	2.29	3	9250	8.12	6
83	*Raphanus sativus oleiformes*	23	8	6	3	5	2	63	43	20	12	19	6	1	5	2	4	9	120	273	44	white	14	170	1.88	108	414	71.34	543
84	*Descurainia sophia*	19	6	5	1	3	0	37	36	1	21	0	2	2	0	2	0	11	78	4650	4	yellow	2	186	1.52	8	1665	4.83	26
85	*Sisymbrium officinale*	14	3	3	1	1	1	15	14	1	1	1	2	1	1	0	1	9	68	5346	14	yellow	4	150	0.60	9	1460	10.53	21
86	*Lepidium campestre*	14	4	4	0	3	0	25	25	0	11	0	0	0	0	0	0	10	25	30020	39	white	2	231	2.67	2	4330	22.43	20
87	*Brassica nigra*	24	9	4	6	7	2	111	49	62	53	19	9	1	8	4	5	6	93	412	28	yellow	7	216	10.78	78	575	35.46	131
88	*Thlaspi arvense*	16	6	4	2	2	2	55	52	3	3	3	3	2	1	1	1	7	29	600	NA	white	4	235	0.33	12	1350	9.25	157
89	*Brassica juncea*	25	12	8	5	9	2	628	529	99	95	23	8	4	4	4	2	6	128	1008	31	yellow	7	136	13.17	76	176	6.93	49
90	*Rapistrum rugosum*	24	11	8	5	7	2	349	334	15	30	5	5	2	3	3	2	8	93	4977	39	yellow	7	469	16.83	73	3650	39.21	21
92	*Arabidopsis thaliana*	5	1	0	1	0	1	2	0	2	0	2	1	1	0	0	0	3	16	1656	36	white	4	15	0.05	0	4450	13.20	13
93	*Thlaspi arvense*	5	3	2	1	0	1	43	41	2	0	2	0	0	0	0	0	2	19	268	NA	white	4	189	0.80	11	1440	17.89	159
94	*Diplotaxis muralis*	11	4	2	2	1	2	41	7	34	1	34	4	0	4	0	4	3	12	121	8	yellow	7	242	2.37	13	688	18.09	66
96	*Lepidium virginicum*	6	3	3	0	1	0	8	8	0	1	0	0	0	0	0	0	3	29	8798	4	white	1	875	0.79	5	5480	7.94	6
97	*Camelina sativa*	8	2	0	2	1	1	2	0	2	1	1	1	0	1	0	1	5	45	547	NA	yellow	7	1060	0.95	19	1300	31.77	37
98	*Sinapis alba*	22	5	4	2	2	1	81	78	3	3	1	4	3	1	0	1	13	67	1144	NA	yellow	12	178	4.22	48	1065	68.57	154
99	*Raphanus sativus oleiformes*	24	8	7	2	2	2	254	223	31	3	31	7	1	6	1	6	9	130	283	NA	white	15	172	0.71	109	804	91.13	374
100	*Sisymbrium officinale*	14	3	3	0	1	0	15	15	0	1	0	0	0	0	0	0	11	66	7968	NA	yellow	5	278	0.81	12	775	13.15	15
